# Acute effects of different types of cannabis on young adult and adolescent resting-state brain networks

**DOI:** 10.1038/s41386-024-01891-6

**Published:** 2024-05-28

**Authors:** Natalie Ertl, Tom P. Freeman, Claire Mokrysz, Shelan Ofori, Anna Borissova, Kat Petrilli, H. Valerie Curran, Will Lawn, Matthew B. Wall

**Affiliations:** 1 Invicro London, Burlington Danes Building, Hammersmith Hospital, Du Cane Road, W12 0NN London, UK; 2 Faculty of Medicine, Imperial College London, Hammersmith Hospital, Du Cane Road, W12 0NN London, UK; 3 Clinical Psychopharmacology Unit, University College London, 1-19 Torrington Place, WC1E 7HB London, UK; 4 Addiction and Mental Health Group (AIM), Department of Psychology, University of Bath, Bath, UK; 5 National Addiction Centre, Institute of Psychiatry Psychology and Neuroscience, King’s College London, London, UK

**Keywords:** Development of the nervous system, Human behaviour

## Abstract

Adolescence is a time of rapid neurodevelopment and the endocannabinoid system is particularly prone to change during this time. Cannabis is a commonly used drug with a particularly high prevalence of use among adolescents. The two predominant phytocannabinoids are Delta-9-tetrahydrocannabinol (THC) and cannabidiol (CBD), which affect the endocannabinoid system. It is unknown whether this period of rapid development makes adolescents more or less vulnerable to the effects of cannabis on brain-network connectivity, and whether CBD may attenuate the effects of THC. Using fMRI, we explored the impact of vaporized cannabis (placebo, THC: 8 mg/75 kg, THC + CBD: 8 mg/75 kg THC & 24 mg/75 kg CBD) on resting-state networks in groups of semi-regular cannabis users (usage frequency between 0.5 and 3 days/week), consisting of 22 adolescents (16–17 years) and 24 young adults (26–29 years) matched for cannabis use frequency. Cannabis caused reductions in within-network connectivity in the default mode (F[2,88] = 3.97, P = 0.022, η² = 0.018), executive control (F[2,88] = 18.62, P < 0.001, η² = 0.123), salience (F[2,88] = 12.12, P < 0.001, η² = 0.076), hippocampal (F[2,88] = 14.65, P < 0.001, η² = 0.087), and limbic striatal (F[2,88] = 16.19, P < 0.001, η² = 0.102) networks compared to placebo. Whole-brain analysis showed cannabis significantly disrupted functional connectivity with cortical regions and the executive control, salience, hippocampal, and limbic striatal networks compared to placebo. CBD did not counteract THC’s effects and further reduced connectivity both within networks and the whole brain. While age-related differences were observed, there were no interactions between age group and cannabis treatment in any brain network. Overall, these results challenge the assumption that CBD can make cannabis safer, as CBD did not attenuate THC effects (and in some cases potentiated them); furthermore, they show that cannabis causes similar disruption to resting-state connectivity in the adolescent and adult brain.

## Introduction

Adolescence is a period of intense brain maturation characterized by ongoing structural and functional changes [1], particularly in the endocannabinoid system, where there is rapid maturational development of corticolimbic neuronal populations [2]. Some evidence has suggested that chronic cannabis use during this critical developmental stage may be associated with alterations in brain connectivity and cognitive impairments [3, 4]. Cannabis use in adolescence is also associated with some educational under-achievement [5], risk of cannabis use disorder [6], and greater likelihood of psychosis later in life [7]. However, our recent observational ‘CannTeen’ study, and cross-sectional analyses, have shown that adolescents may not be as vulnerable as previously feared, and cannabis has similar effects on adolescents and adults in various clinical domains [8], cognitive functions [9], reward processes [10], and brain connectivity [11]. Much of the extant research has investigated adolescent vulnerability to chronic effects, with few papers documenting acute effects in adolescents [12–14].

Resting-state networks (RSN) reflect intrinsic brain connectivity patterns during rest and are associated with various cognitive functions [15–17]. These networks undergo significant changes during adolescence as the brain develops and refines its functional organization [18]. One major change to RSNs in adolescence is the transition from a lattice network (characterised by long average path lengths) to a global small-world network (characterised by discrete clustering and short path lengths) structure [19]. This transition in network characteristics as individuals mature may be linked to changes in the endocannabinoid system, which plays a key role in modulating brain connectivity and synaptic function [20], and potentially influencing neural connectivity, information processing, and the development of mature RSNs. Previous work has shown acute cannabis administration can disrupt RSNs; the Default Mode Network (DMN) and salience network (SAL) appear to be particularly susceptible to cannabis [21, 22]. Understanding how acute cannabis exposure influences RSNs in adolescents is crucial for understanding the consequences of cannabis use during this vulnerable period, however, no previous study has directly compared adolescents to adults.

Striatal networks, including the limbic striatum, play a critical role in reward processing, motivation, and emotion regulation [23]. The striatum undergoes substantial changes throughout adolescence [24], and its connectivity is susceptible to modulation by external factors such as cannabis use [25–28]. Investigating these networks may also illuminate the immediate neural consequences of cannabis use.

Furthermore, the composition of cannabis, specifically the levels of Delta-9-tetrahydrocannabinol (THC) and Cannabidiol (CBD), may influence its effects on brain networks. THC is the primary psychoactive component of cannabis, with euphoric and cognitive-altering properties [29]. In contrast, CBD may modulate the effects of THC [30, 31] with potential therapeutic properties including antipsychotic effects [32] and helping treat cannabis addiction [33]. However, these adaptive properties may only be apparent with high doses of CBD (e.g. 600 mg oral dose [34]); lower doses of CBD (e.g., 8 mg vapourised [35]) may not be effective; there is mixed evidence of CBD’s moderating effects at lower doses [9]. Cannabis which differs in THC and CBD content may have dissociable effects on both cortical [21] and striatal [28] connectivity, with CBD providing potential attenuating effects from the disruption to RSNs caused by THC. Understanding how the presence of CBD may interact with THC in influencing brain-network connectivity can provide valuable insights into the way different types of cannabis influence the adolescent and adult brain.

This study aimed to address these issues by employing fMRI to examine the acute effects of cannabis administration on cortical RSNs and striatal networks in both adolescent and young adult populations. By including participants from different age groups we investigated developmental differences in the acute neural response to cannabis. Given the increasing popularity of cannabis strains with high THC content and the potential moderating effects of CBD, we also explored whether the presence of CBD in cannabis influenced the effects. We hypothesised that: ‘THC’ cannabis will disrupt RSNs while ‘THC + CBD’ may moderate this disruption and that adolescents will be more susceptible to disrupting effects than adults. A placebo-controlled, randomised, double-blind fMRI study was conducted with three treatment sessions where participants inhaled: placebo, ‘THC’ (8 mg/75 kg, zero CBD) or ‘THC + CBD’ (8 mg/75 kg THC & 24 mg/75 kg CBD) cannabis before undergoing a resting-state fMRI scan. Seed-based functional connectivity analyses were carried out to see how cannabis affected cortical and striatal network connectivity in the two age groups.

## Methods

The data derives from the acute-challenge arm of the ‘CannTeen’ study. The full study protocol is available online [36] (https://osf.io/z638r/) and includes further specification of aims, data collection procedures, tasks, and power calculations. This study was not a clinical trial under the definition of the UK Medicines and Health Care Products Regulatory Agency; however, it was registered on clinicaltrials.gov (April 20, 2021, ID = NCT04851392; https://www.clinicaltrials.gov/study/NCT04851392). The analysis plan for the data presented here was also pre-registered (https://osf.io/s5vz8) prior to any analysis taking place.

### Participants

Participants were 48 current (semi-regular) cannabis users with usage frequency between 0.5 and 3 days/week averaged over the past 3 months. Participants were able to have used other illicit drugs, but they were excluded if this use exceeded more than twice per month. There was an equal split of 24 adults (mean age 27.8 years, 12 females) and 24 adolescents (mean age 17.2 years, 12 females). Participants were recruited from the Greater London area via school assemblies, physical posters and flyers, and online (Facebook, Instagram, and Gumtree) advertisements. For the full inclusion/exclusion criteria, see the main CannTeen study protocol [36]. This was a per-protocol study, therefore subjects who dropped out were replaced to ensure that 48 subjects completed all three study sessions. Further information on participant characteristics is in the supplementary material.

### Procedure

Participants completed three drug administration and MRI scanning sessions at the Invicro clinical imaging facility, Hammersmith Hospital, London, UK. Sessions were conducted between 11th March 2019, and 16th June 2021. First, participants completed instant saliva drug (Alere DDSV 703 or ALLTEST DSD-867MET/C) and breathalyser (Lion Alcometer 500) tests, as well as self-reported abstinence, to confirm no use of alcohol in the previous 24 h and no use of cannabis or other illegal drugs in the previous 72 h.

Cannabis (dried medical cannabis flower) was sourced from Bedrocan (Netherlands) and imported into the UK under a Schedule 1 Home Office License. The cannabis was administered with a Volcano Medic Vaporizer (Storz and Bickel) set at 210 °C. Three types of cannabis were used to create the formulations: Bedrocan (20.2% THC, 0.1% CBD), Bedrolite (0.4% THC, 8.5% CBD), and Bedrobinol (no THC or CBD). There was an absence of microbes, yeasts, aflatoxins, pesticides, and heavy metals in both Bedrolite and Bedrobinol and there was the presence of cannabinol at 0.1%. Appropriate quantities of these three cannabis types were combined to produce the following treatments, matched for overall weight of cannabis: 0.107 mg/kg THC in the “THC” condition (e.g. 8 mg THC for a 75 kg person), 0.107 mg/kg THC plus 0.320 mg/kg CBD in the “THC + CBD” condition (e.g. 24 mg CBD for a 75 kg person), or placebo cannabis (0 mg THC, 0 mg CBD). The dose of THC used was equivalent to 1.6 standard units of THC [37]. Subjects inhaled two balloons (each within nine minutes, a total of 18 min), with the experimenters monitoring standard timings for inhalation. This method of administration has been extensively used in previous work [12, 35, 38], and is safe and effective at delivering cannabinoids and producing behavioural and subjective effects. Placebo cannabis was closely matched to the active drug conditions in both appearance and smell, and all researchers present (as well as the participant) were blinded to the drug conditions. Additional staff (not present at the testing sessions) blinded the treatment conditions in advance of the testing sessions. The minimum washout period between testing sessions was three days, the mode was seven days, and the maximum was 51 days.

The resting-state scan was eight minutes long and was acquired towards the beginning of the scanning session, after the anatomical scans, and a stop-signal task (reported elsewhere). The resting-state scan therefore occurred approximately 50 min after the start of drug administration. Previous work has shown that subjective effects of vaporized cannabis have a fast onset, and stay at a high level for approximately 60–90 min [39]. The timing of the resting-state scan was therefore likely close to the time of peak effects. Participants were instructed to keep their eyes open but blink as normal during the scan in order to mitigate against them falling asleep.

### MRI data acquisition

MRI data were collected using 3.0 T Siemens Magnetom Verio and Siemens Magnetom Trio scanners, both using 32-channel phased-array head coils. Thirty-six subjects were scanned on the Verio, and 12 were scanned on the Trio (subjects always completed all three testing sessions on the same scanner). Settings used for the acquisition sequences were identical on both scanners. For more information on MRI data acquisition see Supplementary Material.

### Analyses

Demographic, drug use, and mental health data were compared between the adolescent and adult groups using unpaired *t*-tests or chi-squared tests as appropriate. All resting-state fMRI analysis procedures broadly followed those used in previous work [11, 21, 28, 40].

#### Preprocessing and first-level analyses

fMRI analyses used FSL (FMRIB Software Library v6.0, Analysis Group, FMRIB, Oxford, UK) with standard preprocessing pipelines including brain extraction, head motion correction, temporal filtering, and spatial smoothing (6 mm FWHM Gaussian kernel); for more detail see Supplementary Material. Participants were excluded if they exceeded >3 mm movement in any direction and >1 mm mean displacement. The mean movement was then compared across age and drug groups to check for any significant differences which may bias the data; see supplementary material.

Seed-based functional connectivity methods were used. This analysis method uses a time-series from a particular region (the ‘seed’) to identify other brain areas that have correlated time-series; the implication being that areas with similar temporal characteristics are functionally connected. The seeds selected reliably define a network. For ease of narrative, we will be referring to connectivity with a network, however, in strict terms, we are referring specifically to connectivity with the seed.

Four cortical resting-state networks were investigated. The seeds used to define these networks were:

- The posterior cingulate (PCC) to define the Default Mode Network (DMN)
- The anterior insula to define the salience network
- The dorsolateral prefrontal cortex (DLPFC) to define the Executive Control Network (ECN)
- A hippocampal seed to define the hippocampal network

See Supplementary Fig. 2A for the image showing seeds. The regions for the PCC and anterior insula seeds were the same as those used in [21]. These were derived from automated meta-analytic data on http://neurosynth.org/ using the ‘default mode’ and ‘salience’ terms (uniformity tests). We used a region in the DLPFC as recommended by [17] as the seed region for the ECN, which was also derived from http://neurosynth.org/, using the “executive control” term. These meta-analysis maps were thresholded at an appropriate level (Z = 12/10/6 for the default mode, salience, and executive control maps, respectively) to achieve anatomically plausible regions. The PCC, anterior insula and DLPFC clusters were then isolated and binarized for use as image masks. The hippocampus seed region was defined anatomically using the Harvard-Oxford subcortical atlas.

Three striatal networks were also investigated. These were the:

- Associative (including caudate head and putamen)
- Limbic (including nucleus accumbens and ventral caudate)
- Sensorimotor (including putamen tail)

Masks for the three striatal networks (associative, limbic, and sensorimotor) were the same as those used in [11, 28] and are defined according to the original parcellation by [41] and [42], using the atlas provided by [43]. The associative mask includes the precommissural dorsal caudate, the precommissural dorsal putamen, and the postcommissural caudate. The limbic mask includes the ventral caudate and substantia nigra, and the sensorimotor mask comprises the postcommissural putamen (see Supplementary Fig. 2B and Supplementary Table 2 for centre of gravity coordinates for each seed).

The seven standard space mask images were co-registered to each subject’s functional space, thresholded at 0.5, and binarised to produce the final individualised mask images. Mean time-series from these masks were used in first-level analysis models as regressors of interest, with white matter (WM) and cerebrospinal fluid (CSF) regressors added to the model as noise regressors (as used previously [11, 21, 28]), along with an extended set of head motion parameters. WM and CSF regressors were generated in a similar manner to the seed-masks, for more information on the generation of WM and CSF regressors please see supplementary material.

All second-level analyses used FMRIB’s local analysis of mixed effects (FLAME); a two-step process using Bayesian modelling and estimation, with a weighted least-squares approach which does not assume equal variance between groups. All group-level analyses used cluster-level thresholding [44, 45] with a cluster-defining threshold of *Z* = 2.3 and a multiple-comparisons corrected cluster-extent threshold of *P* < 0.05, in order to account for multiple comparisons. Following convention, and previous similar work [11, 21, 28, 40], brain networks were treated as conditionally independent [46], and therefore no additional correction for the number of networks was applied.

##### Within-network connectivity (network ROI analysis)

Initially, a group-mean (entire sample mean) analysis was performed collapsing across subjects and cannabis types; the resulting networks were validated against previous studies [21, 28]. Network masks were created from this analysis and used to extract parameter estimates representing overall connectivity *within* each network. Maps were thresholded at *Z* = 80% of the maximum voxel value to define a plausibly anatomically constrained set of regions; the threshold levels are outlined in supplementary table 3. The thresholded network maps were then binarized to produce masks, from which data could be extracted to give estimates of overall network differences in the groups and drug conditions. These estimates (single values, representing overall connectivity within the network) were analysed using 2 × 3 ANOVA models to test for effects of age (adolescent vs. adult), cannabis type (placebo, THC, THC + CBD), and any interaction.

The extracted parameter estimates from each network were also correlated with cannabis use frequency (days cannabis was used per week in the last three months) The alpha threshold for these correlations was reduced to 0.007 to reflect the seven tests (across the seven networks) conducted. These analyses were conducted using *Jamovi version 2.3.21.0*.

##### Seed-voxel (whole-brain) analysis

Next, to investigate the main effect of the drug and the drug*age-group interaction in a voxel-wise, whole-brain manner, a 3 × 2 mixed measures ANOVA model was constructed. F tests were used to reveal significant differences between groups, and significant interaction effects. Analyses of this type produce maps of F statistics which (unlike t statistics used for simple contrasts) are non-directional (always positive) and are therefore uninformative as to the direction of the effects. Therefore, the significant clusters resulting from these ANOVA analyses were defined as ROIs and mean values were extracted from these regions for each participant (shown in Supplementary Fig. 7). These values were then plotted to determine the precise pattern and direction of the effects across the three cannabis conditions and two age groups. *Post hoc* t-tests were conducted and resulting p-values were Tukey corrected.

##### Age effects

Finally, to test for age main effects, mid-level fixed-effects analyses were performed. These models averaged across all cannabis conditions for each participant, resulting in a single mean map for each individual. These mid-level means were then used in simple unpaired *t*-test models to assess the main effect of age on network connectivity.

As a precautionary measure, all analysis models were rerun with an additional regressor which modelled the scanner (Verio/Trio) used for that subject.

## Results

### Head motion

Two participants (both in the adolescent group) exhibited head motion of >3 mm in at least one scan and were excluded, leaving a final sample of N = 46 (22 adolescents, 24 adults). Head motion was analysed in the remaining participants by investigating: mean framewise displacement, total displacement, and number of outlying volumes. No significant effect of cannabis treatment was found in any measure, suggesting head motion was similar across all treatment conditions. For further information see supplementary materials.

### Participants

A summary of the participant characteristics (demographics, questionnaire scores, and drug history) can be found in Table 1.

**Table 1:** Demographic, questionnaire and drug history information of adolescent and adult participants.

	Adolescent	Adult	Group differences
	(*n* = 22)	(*n* = 24)	
Gender, n (%)			*χ*^2^(1, N = 46) = 0.00, P = 1
Female	11 (50%)	12 (50%)	
Male	11 (50%)	12 (50%)	
Age in years, mean (SD)	17.2(0.44)	27.8 (1.04)	*** Adult > Adolescent t[44] = −44.4, P < 0.001
Maternal education, n (%)			*χ*^2^(1, N = 46) = 0.047, P = 0.83
Below undergraduate degree	8 (36%)	8 (33%)	
Undergraduate degree or above	14 (64%)	16 (67%)	
Education, degree level and above			
* Adults only*	N/A	19 (79%)	*N/A*
BDI, mean (SD)	8.7 (6.91)	5.3 (8.52)	t[44] = 1.77, P = 0.084
Use of alcohol every week, n (%)			*** Adult > Adolescent *χ*^2^(1, N = 46) = 12.5, P < 0.001
No	17 (77%)	6 (25%)	
Yes	5(23%)	18 (75%)	
Use of cigarette/roll-ups every week, n (%)			*χ*^2^(1, N = 46) = 0.71, P = 0.40
No	15 (68%)	19 (79%)	
Yes	7 (32%)	5 (21%)	
Other illicit drug use, monthly use, n (%)			*χ*^2^(1, N = 46) = 0.27, P = 0.60
No	21 (95%)	22 (92%)	
Yes	1 (5%)	2 (8%)	
Cannabis use			
Weekly, n (%)			*χ*^2^ (1, N = 46) = 0.46, P = 0.50
No	2 (9%)	1 (4%)	
Yes	20 (91%)	23 (96%)	
Cannabis frequency (dpw) mean, SD	2.55 (1.01)	2.79 (1.10)	t[44] = 0.787, P = 0.44
Hours since last use (users), mean (SD) [min-max]^c^	196(182)	128(62.4)	t[44] = 1.71, P = 0.09
Age of first ever use, mean (SD)	14.7 (0.93)	18.2 (2.62)	*** Adult>Adolescent t[44] = -5.90, P < 0.001
Premorbid IQ (WTAR) mean (SD)	112 (12.1)	118 (6.12)	*Adult>Adolescent t[44] = −2.18, P = 0.035
CUDIT, mean (SD)	9.95 (3.17)	7.21 (3.31)	**Adolescent>Adult t[44] = 2.87, P = 0.006
DSM-5 severe CUD (users), n (%)			*χ*^2^(1, N = 46) = 1.12, P = 0.29
No	21 (96%)	24 (100%)	
Yes	1 (4%)	0 (0%)	

Sociodemographic characteristics of the full sample minus the two subjects excluded for head motion (final n = 46). BDI is the Beck Depression Inventory. WTAR is Wechsler’s Test of Adult Reading. CUDIT is the Cannabis Use Disorder Inventory Test. Continuous data are presented as mean [SD], and categorical data are presented as n (%). Group differences are highlighted in the final column using appropriate tests for each data type (*χ*^2^ and t-tests; *P < 0.05, **P < 0.01, ***P < 0.001).

### Within-network connectivity (network ROI analysis)

The entire sample mean networks closely match previous work [21, 28] and therefore validate the general acquisition and analytic approach and procedures. These are shown in supplementary figure 3, the minimum cluster thresholds for each network are shown in Supplementary Table 3, along with the maximum Z value of each analysis which was used to threshold the network maps and make the subsequent network masks, shown in Supplementary Fig. 4.

**Fig. 1:**
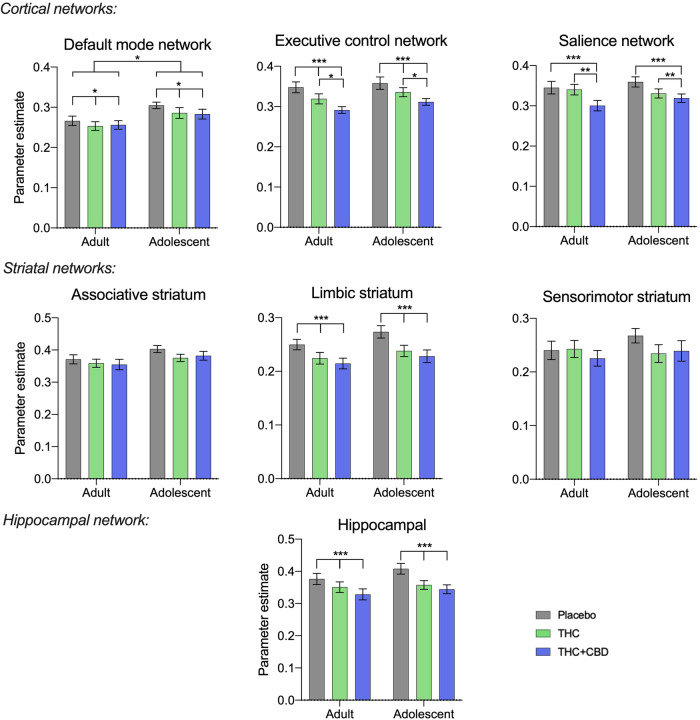
Summary values of connectivity strength across entire networks. Values are means, and error bars show SEM, N = 46 (24 adults), *P* < 0.001 ***, P < 0.01**, P < 0.05 *.

Analysis of within-network connectivity investigating the effects of age, cannabis type, and interaction effects found significant effects of cannabis type in most networks (excluding the associative and sensorimotor striatal networks). Significant effects of age were found in the DMN, but no interaction effects were observed. The ANOVA effects are summarised in Supplementary Table 4 and the *post hoc* results in Supplementary Table 5, with summary graphs in Fig. 1.

Acute cannabis administration (both ‘THC’ and ‘THC + CBD’) reduced overall connectivity in all the cortical networks, plus the hippocampal network, relative to placebo. Network connectivity is further significantly reduced in the salience network and the ECN with ‘THC + CBD’ compared to ‘THC’ alone. In the striatal analyses, only the limbic striatal network was affected by acute cannabis administration. Adolescents had significantly greater network connectivity in the DMN than adults. There were no significant interaction (age*drug) effects. The present results were not significantly affected by the scanner used, see Supplementary Fig. 5.

#### Correlations

There were no correlations between cannabis use frequency (how many times cannabis was used per week in the last three months) and effects of cannabis (i.e. THC vs. placebo and THC + CBD vs. placebo) on whole network connectivity.

### Seed-voxel (whole-brain) analyses

#### Main effect of cannabis

Significant effects of the cannabis type (placebo, ‘THC’, or ‘THC + CBD’) were found on network connectivity within the ECN, salience network, limbic striatal network and hippocampal network. These results are F statistics derived from the 2 × 3 ANOVA model and so are directionless and not informative about exactly which drug conditions show significant differences. These main effects of cannabis treatment are shown in Supplementary Fig. 6 and a table showing the minimum cluster size threshold for each network analysis is shown in Supplementary Table 6.

To further investigate the precise pattern and direction of the results identified in these F statistic maps, the results were subdivided into Regions of Interest (ROIs) and data were then extracted to compare the relative connectivity between age groups in each drug condition. These ROIs are shown in Supplementary Fig. 7.

#### Cortical resting-state networks

**Fig. 2:**
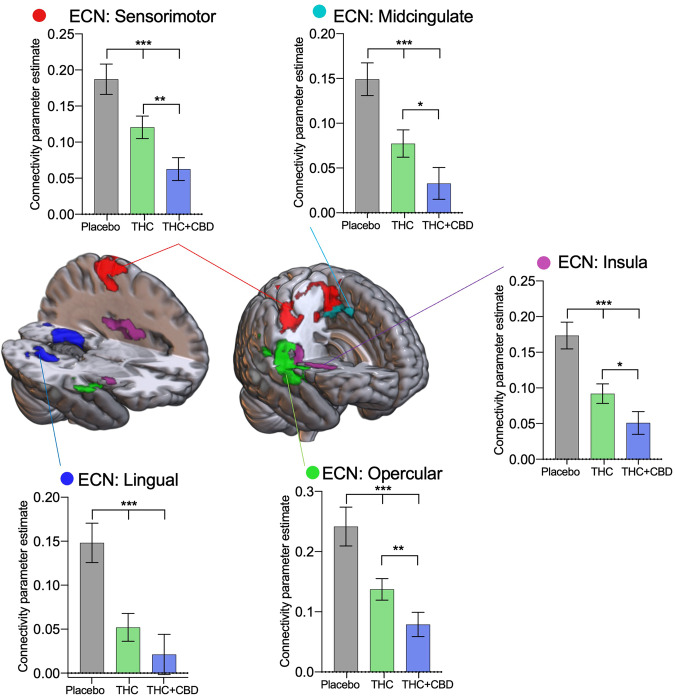
Drug treatment results from the Executive Control Network (ECN). Areas identified in a seed-voxel whole-brain ANOVA model as having connectivity affected by drug condition (placebo, THC, THC + CBD) with the Executive Control Network (ECN), were subdivided into regions of interest to identify directional effects. Connectivity between the ECN and the sensorimotor cortex (red), midcingulate (light blue), insula (purple), opercular cortex (green), and lingual gyrus (dark blue) is significantly reduced with acute cannabis administration. Results are presented collapsed across age groups. Error bars show SEM, N = 46 (24 adults), *P* < 0.001 ***, P < 0.01**, P < 0.05 *.

The main cannabis effects found in the ECN are outlined in Fig. 2. No main effects of age or interaction were found so results are presented collapsed across age groups. Summary statistics can be found in Supplementary Table 7, and a non-collapsed across the group-level figure in Supplementary Fig. 8. Overall a reduction in connectivity was observed in both cannabis treatments compared to placebo. Connectivity was reduced between the ECN and the sensorimotor cortex (F[2,88] = 19.57, P < 0.001). *Post hoc* tests revealed THC (t[44] = 3.33, P = 0.005) and THC + CBD (t[44] = 5.24, P < 0.001) significantly reduced connectivity between the ECN and the sensorimotor cortex, and THC + CBD reduced connectivity significantly more relative to THC alone (t[44] = 3.87, P = 0.001). In the midcingulate (F[2,88] = 20.84, P < 0.001) *post hoc* tests revealed THC (t[44] = 4.05, P < 0.001) and THC + CBD (t[44] = 5.71, P < 0.001) significantly reduced connectivity between the ECN and the midcingulate relative to placebo, and the reduction caused by THC + CBD was significantly greater than THC alone (t[44] = 2.73, P = 0.024). Similarly, connectivity with the insula (F[2,88] = 27.29, P < 0.001), was significantly lower in the THC condition (t[44] = 4.75, P < 0.001) and the THC + CBD condition relative to placebo (t[44] = 6.48, P < 0.001), while THC + CBD also reduced connectivity significantly relative to the THC alone condition (t[44] = 2.86, P = 0.017). Connectivity with the opercular cortex (F[2,88] = 18.20, P < 0.001) was significantly altered between all three drug conditions, placebo > THC (t[44] = 3.58, P = 0.002), placebo>THC + CBD (t[44] = 5.04, P < 0.001), THC > THC + CBD (t[46] = 3.12, P = 0.009). Finally, connectivity with the lingual gyrus (F[2,88] = 12.11, P < 0.001) was significantly reduced by both THC (t[44] = 3.83, P0.001) and THC + CBD (t[44] = 4.19, P < 0.001).

**Fig. 3:**
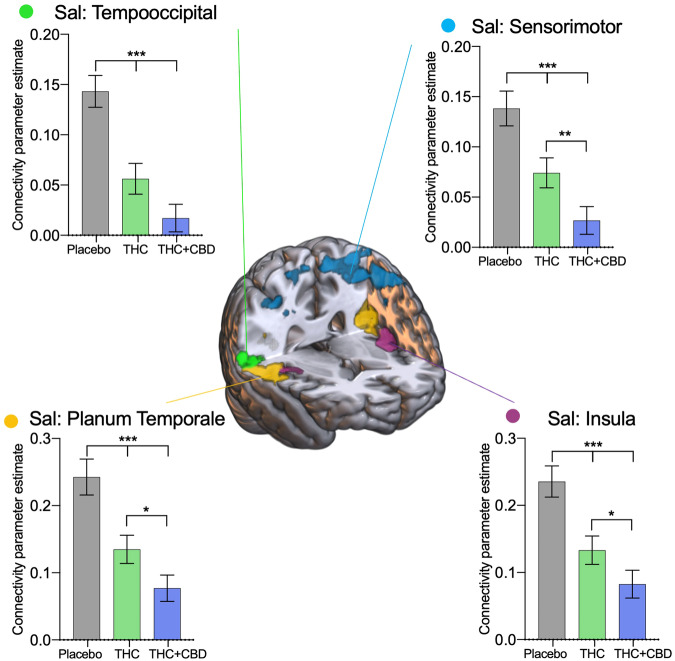
Drug treatment results from the salience network. Areas identified in a seed-voxel whole-brain ANOVA model as having connectivity affected by drug condition (placebo, THC, THC + CBD) with the salience network (Sal), were subdivided into regions of interest to identify directional effects. Connectivity between the salience network and the temporooccipital cortex (green), sensorimotor cortex (blue), planum temporale (yellow), and insula (purple) are significantly reduced with acute cannabis administration. Results are presented collapsed across age groups. Error bars show SEM, N = 46 (24 adults), *P* < 0.001 ***, P < 0.01**, P < 0.05 *.

The acute cannabis effects on the salience network are outlined in Fig. 3, no main effects of age or an interaction were found, so results are presented collapsed across age groups. Summary statistics can be found in Supplementary Table 8, and a non-collapsed across the group-level figure in Supplementary Fig. 9. Cannabis administration reduced connectivity between the salience network and all the defined ROIs. Specifically, a bilateral area around the temporooccipital cortex (F[2,88] = 27.05, P < 0.001), was significantly altered by THC (t[44] = 4.92, P < 0.001) and THC + CBD (t[44] = 7.14, P < 0.001) relative to placebo. Connectivity with the salience network and the sensorimotor cortex (F[2,88] = 20.12, P < 0.001), was significantly reduced with THC (t[44] = 3.73, P = 0.002) and THC + CBD (t[44] = 3.06, P < 0.001), relative to placebo. The THC + CBD administration reduced connectivity with the sensorimotor cortex significantly compared to THC alone (t[44] = 5.61, P = 0.010). The insula (F[2,88] = 20.60, P < 0.001) also significantly reduced connectivity with the salience network with THC (t[44] = 4.06, P < 0.001) and THC + CBD (t[44] = 5.57, P < 0.001) administration relative to placebo. THC + CBD administration also reduced connectivity with the insula significantly compared to THC alone (t[44] = 2.61, P = 0.032). Finally in an area around the planum temporale cortex (F[2,88] = 20.87, P < 0.001), THC (t[44] = 4.02, P < 0.001) and THC + CBD (t[44] = 5.07, P < 0.001) significantly reduced connectivity relative to placebo, and THC + CBD reduced connectivity significantly more than THC alone (t[44] = 2.67, P = 0.028).

There were no acute cannabis effects on connectivity in the analyses of the DMN.

#### Striatal networks

**Fig. 4:**
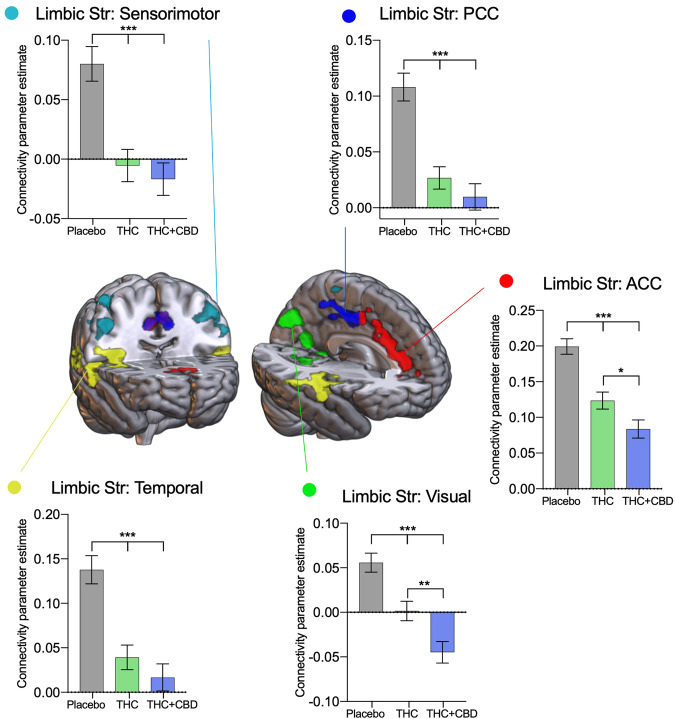
Drug treatment results from the limbic striatal network. Areas identified in a seed-voxel whole-brain ANOVA model as having connectivity affected by drug condition (placebo, THC, THC + CBD) with the limbic striatal network, were subdivided into regions of interest to identify directional effects. Connectivity between the limbic striatal network and the sensorimotor cortex (light blue), posterior (dark blue) and anterior (red) cingulate, visual cortex (green), and temporal cortex (yellow) are significantly reduced with acute cannabis administration. Results are presented collapsed across age groups. Error bars show SEM, N = 46 (24 adults), *P* < 0.001 ***, P < 0.01**, P < 0.05 *.

Five ROIs were identified as having connectivity with the limbic striatum affected by acute cannabis administration (Fig. 4), no main effects of age or interaction were found, so results are presented collapsed across age groups, and summary statistics can be found in Supplementary Table 9, and a non-collapsed across the group-level figure in Supplementary Fig. 10. A bilateral reduction in connectivity was observed with an area around the sensorimotor cortex (F[2,88] = 23.96, P < 0.001) after THC (t[44] = 5.12, P < 0.001) and THC + CBD administration (t[44] = 5.66, P < 0.001) relative to placebo. Connectivity between the posterior cingulate and the limbic striatum was significantly reduced with cannabis administration relative to placebo (F[2,88] = 35.84, P < 0.001), with both THC (t[44] = 5.71, P < 0.001) and THC + CBD (t[44] = 8.04, P < 0.001) treatments. Similarly the anterior division of the cingulate (F[2,88] = 35.84, P < 0.001) had reduced connectivity with both THC (t[44] = 5.71, P < 0.001) and THC + CBD (t[44] = 8.04, P < 0.001) relative to placebo. THC + CBD also significantly reduced connectivity compared to THC alone (t[44] = 2.88, P = 0.017) in this region. An area in the visual cortex (F[2,88] = 20.94, P < 0.001) had reduced connectivity with the limbic striatum with THC administration relative to placebo (t[44] = 3.58, P = 0.002); this connectivity was reduced further with THC + CBD administration (t[44] = 6.31, P < 0.001) relative to placebo. The reduction was so great that there was a significant difference in connectivity between the THC and THC + CBD conditions (t[44] = 2.96, P = 0.013). The final ROI identified as having connectivity with the limbic striatum significantly altered with cannabis administration was a bilateral area around the temporal lobes (F[2,88] = 26.47, P < 0.001); both THC (t[44] = 5.57, P < 0.001) and THC + CBD (t[44] = 5.71, P < 0.001) significantly reduced connectivity compared to placebo.

There were no effects of acute cannabis administration on connectivity with the associative or sensorimotor striatum seed regions.

#### Hippocampal network

**Fig. 5:**
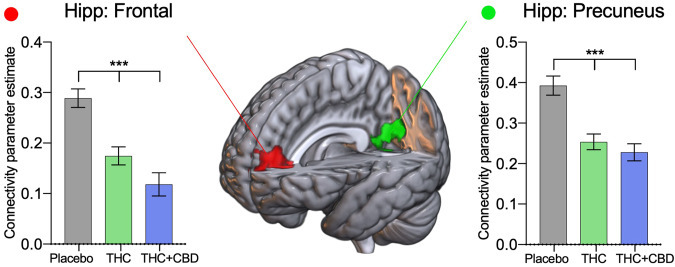
Drug treatment results from the hippocampal network. Areas identified in the seed-voxel whole-brain ANOVA model as having connectivity affected by drug condition (placebo, THC, THC + CBD) with the hippocampal network were subdivided into regions of interest to identify directional effects. Connectivity between the hippocampal network and the frontal (red) and precuneus cortex (green) is reduced with acute cannabis administration. Results are presented collapsed across age groups. Error bars show SEM, N = 46 (24 adults), *P* < 0.001 ***.

Two distinct regions were identified as having significantly altered connectivity with the hippocampus (Fig. 5). A medial frontal cortex region showed significantly reduced connectivity (relative to placebo): (F[2,88] = 22.64, P < 0.001), THC (t[44] = 4.40, P < 0.001) and THC + CBD (t[44] = 4.91, P < 0.001). The second region around the precuneus (F[2,88] = 25.66, P < 0.001), where THC (t[44] = 5.54, P < 0.001) and THC + CBD (t[44] = 7.18, P < 0.001) significantly reduced connectivity with the hippocampus. No main effects of age or interaction were found, so results are presented collapsed across age groups, summary statistics can be found in Supplementary Table 10, and a non-collapsed across the group-level figure in Supplementary Fig. 11.

None of the results were significantly affected by the scanner used, see Supplementary Fig. 12.

### Age main effects

Significant effects of age were found in two resting-state networks (executive control and salience) and two striatal networks (associative and limbic). These are summarised in supplementary figure 13. Generally, there was greater connectivity with regions in the cortex in adolescents compared to adults. These most plausibly represent developmental effects.

### Subjective effects

Changes in psychological/subjective effects in this cohort have been reported elsewhere [9]. Subjects could identify when on placebo vs cannabis, however, there were no differences in the subjective effects reported for ‘THC’ and ‘THC + CBD’ cannabis.

## Discussion

Acute cannabis administration reduced within-network connectivity in five of the seven networks tested. ‘THC + CBD’ tended to reduce this connectivity to a greater degree than ‘THC’ alone, however, this effect was only significant in the ECN and the salience network. Interestingly, within-network connectivity in the associative and sensorimotor striatum was unaffected by either strain of cannabis.

In the seed-voxel analyses, four networks were significantly altered with cannabis administration relative to placebo. These were two cortical resting-state networks (ECN and salience network), one striatal network (limbic), and the hippocampal network. In these analyses again the general pattern was that the ‘THC + CBD’ treatment reduced connectivity more than THC alone.

Importantly, there were no age * drug interaction effects, suggesting that age does not significantly change the way cannabis disrupts RSN connectivity. This was contrary to our hypothesis, however, this lack of interaction effect matches other data collected in this trial. For example, no interaction effects have been found on reward anticipation [27], acute psychotomimetic, verbal memory-impairing, or subjective effects of cannabis [9], and when comparing matched groups of adult/adolescent and regular users/non-users in a cross-sectional study of RSN connectivity [11].

THC is primarily responsible for the psychoactive effects of cannabis [47] while some previous work suggests that CBD may possess therapeutic and neuro-protective properties and the ability to modulate the negative effects of THC [21, 48]. CBD is a negative allosteric modulator of the CB1R [49], meaning it reduces the binding affinity of THC to the CB1 receptor, and so dampens the intensity of THC’s effects. Previous work has shown the addition of CBD reduced the disruption caused by THC to RSN connectivity [21]. However, the findings of the present study (that added CBD in fact produces greater reductions in connectivity than THC alone) are consistent with other recent research [9, 50]. Both THC and CBD undergo metabolism facilitated by cytochrome P450 enzymes [51]. Consequently, when CBD is administered in higher doses, it competes with these metabolic enzymes, resulting in reduced THC metabolism [51]. This competition paradoxically leads to elevated plasma THC levels when THC and CBD are co-administered. CBD also inhibits the activity of cytochrome P450 enzymes, thereby amplifying this effect [52]. Increased plasma THC with CBD (pre) administration has been demonstrated convincingly in animal research [53, 54]. The increased plasma THC concentration may, in part, account for the heightened connectivity disruption observed here in the ‘THC + CBD’ condition. The research into the moderating effects of CBD on acute THC administration is overall mixed, for a comprehensive systematic review please see [55]. It is likely that the relative THC and CBD doses are pivotal for their effects on brain connectivity, perhaps with distinct mechanisms (potentially mediated by cytochrome P450 activity) prevailing at different doses.

We previously identified differences in connectivity between cannabis users and non-using controls in the ECN [11] in a cross-sectional study of resting-state connectivity in cannabis users compared to controls (i.e., *not* under acute cannabis exposure) which used similar analysis methods. Five regions (motor cortex, cingulate, insula, posterior temporal parietal junction and the superior temporal gyrus) were identified as having *increased* connectivity with the ECN in the cannabis user group. These areas are very similar to the regions identified in the present study (sensorimotor area, insula, cingulate, opercular cortex and lingual gyrus), as having *decreased* connectivity with the ECN with acute cannabis administration. This data therefore supports our proposal from [11] which suggested increases in ECN connectivity in cannabis users relative to controls may be a compensatory mechanism to support the reductions in connectivity produced by regular cannabis intoxication.

The findings are also somewhat consistent with other previous acute-challenge cannabis studies. These have also found strong effects in the salience network [21], and the limbic striatum [28]. A recent mega-analysis study (aggregating data from three previous acute-challenge studies, total N = 87) highlighted the salience network, with minimal effects seen in the DMN and ECN, and effects in subcortical networks significantly modulated by COMT genotype [56]. Previous work has also identified significant effects on the DMN [57], which we find only minimal evidence of here. A recent novel, whole-brain approach has demonstrated widespread hypoconnectivity effects with cannabis intoxication, most prominently in subcortical, limbic, and attentional networks [58]. This may suggest that the effects of cannabis intoxication are perhaps not specific to particular brain networks, and may be analogous to the more global effects on brain connectivity seen with classic psychedelics [59–61].

Of the four networks which showed altered connectivity with cannabis administration, all except for the hippocampal network showed a significant reduction in connectivity with the sensorimotor cortex. The sensorimotor cortex has a high concentration of CB1 receptors [62] and sensorimotor effects are a common side effect of acute cannabis administration (e.g. enhanced pleasure [63] and reduced driving ability [64]). Diminished motor coordination and sensorimotor integration are symptoms which have been reported in individuals with high levels of cannabis use [65]. It is therefore of interest that connectivity with the sensorimotor cortex was reduced with these RSNs and the limbic striatal network, but *not* the sensorimotor striatum.

Cannabis reduced cingulate connectivity in the ECN and limbic striatal network. The ECN, crucial for attention, working memory, decision-making, and cognitive control, relies on the anterior cingulate cortex [66]. Acute cannabis disrupted this connectivity, potentially disrupting attention, memory, and decision-making [67, 68]. The cingulate cortex is also connected to the nucleus accumbens (part of the limbic striatum), regulating emotion and motivation [69]. Disruptions may impact emotional processing and reward sensitivity. Using a monetary reward task, we have also previously demonstrated significant attenuating effects of cannabis on reward anticipation activity in the limbic striatum, in the same cohort [27]. However, a similar task showed only minor effects in a study of regular cannabis users and controls [10], suggesting that these acute effects may not persist in the non-intoxicated state.

Reduced connectivity with the insula was identified in both the salience network and the executive control network analyses. The insula is involved in various cognitive and affective processes, including salience detection and integration, interoceptive awareness, and emotion regulation [70]. The salience network, which includes the insula, is responsible for identifying relevant stimuli and orchestrating appropriate responses [71]. Studies have suggested that the salience network is the mediator between the ECN and the DMN [72], making the salience network vital for interacting with the external world. Previous studies have drawn a connection between cannabis use, disrupted salience processing, and cannabis-induced psychosis [21, 73]. This disruption may result in aberrant salience processing and altered integration of interoceptive signals, potentially contributing to cognitive deficits and disrupted emotional regulation associated with cannabis use [74].

The hippocampus plays a crucial role in memory formation and retrieval. Our data showed acute cannabis administration reduced fronto-hippocampal and precuneus-hippocampal connectivity. Reduced connectivity between the frontal cortex and the hippocampus due to cannabis use may lead to difficulties in cognitive domains and may be relevant to the common side effects experienced with acute cannabis administration such as impaired memory, reduced cognitive flexibility, decreased attentional control, and altered decision-making processes [75]. Hippocampal-precuneus connections are important for episodic autobiographical memory [76], and so reductions here may also contribute to the aberrant memory capacity experienced under acute cannabis administration.

Some differences between the adolescent and adult groups were identified in the ECN, salience network, associative and limbic striatal networks. The most plausible interpretation of these effects is that they are developmental in origin. The most robust age difference was observed in the limbic striatal network, where adolescents tended to have greater connectivity in regions associated with the DMN. Since the limbic striatum is involved with addiction (and cannabis use disorder) and the DMN with introspection, this increased connectivity may reflect the inflated chances of adolescents for developing a cannabis use disorder, which is three times the rate of adults [8]. This finding may be important since we identified a significant difference between the adolescents and adults on the cannabis use disorder inventory test (CUDIT) scores, suggesting the adolescent group may be slightly more vulnerable to cannabis use disorders than adults, though neither group met the threshold for potential CUD.

The findings of this study have two key implications for public health. Firstly, our findings that CBD did not attenuate the effects of THC (and conversely potentiated them in some cases) challenge the assumption that CBD can reduce the harms of cannabis. Given that public health guidelines for lower-risk cannabis use state it is advisable to use cannabis containing high CBD:THC ratios [77], our findings suggest an important need to communicate to potential consumers that CBD may not attenuate (and in fact may exacerbate) the effects of THC. Secondly, we found similar effects of THC in disrupting resting-state connectivity across a range of networks, when comparing adolescents with young adults.

### Strengths and limitations

This study had a number of strengths including a relatively large sample size compared to previous similar studies, and an equal split between adolescents and adults. We included equal numbers of both male and female participants, increasing the generalisability of our results, and we also had an appropriate placebo control treatment matched for sensory characteristics. We explicitly matched the cannabis use frequency of adolescents with that of adults, improving on a previous acute study which found significant age differences with adolescents using cannabis more heavily than adults [12], though we cannot extrapolate our results beyond people who use fortnightly–three days per week. Moreover, we closely followed our pre-registered analysis plan which increases scientific rigour, and quality of evidence, and ensures transparency of our data and analyses. Having an even larger sample size would have allowed us to look for sex differences, which in the current study we were not powered to do. We found a small difference in premorbid IQ between the adult and adolescent groups, which may have impacted our results. However, this matches previous research which suggests children (at around age 12) with lower IQ are more likely to become cannabis users during adolescence, but cannabis use in adolescents is not likely to cause a decline in IQ [78]. It is possible that younger adolescents with less developed resting-state brain systems may have shown differential effects to adults, however, there are major ethical considerations with conducting acute drug challenge studies in younger age ranges (<16 years old). Our samples were adolescents (age 16–17), and young adults (age 26–29), however, we excluded adults who were regular users of cannabis before the age of 18. This was done in order to exclude adults who had used cannabis regularly in this key developmental window, however, it means that there is a difference in the age of onset of use in the two groups. In part, this difference is intimately related to the study aims (comparing adolescents with adults) and the nature of cross-sectional comparisons, however, we cannot rule out that this difference may have broader implications (e.g. people who begin using cannabis in adolescence may represent a somewhat different cohort to those who begin using in their 20 s, in a number of possible ways). The restricted age range in the adult group was also used in order to better match the (necessarily) restricted range of the adolescent group, however, this means the findings may not be readily generalisable to older adults. Another potential confounder is the potential effects of cannabis use on myelination, which can affect resting-state network connectivity. Previous work shows that the early onset of cannabis use is correlated with greater disruptions in WM tracts [79]. Moreover, several studies have identified poorer WM integrity with chronic cannabis use [80, 81]. However, the evidence concerning the medicinal uses of cannabis seems to suggest the opposite, with improvements in WM integrity after six months of medicinal cannabis treatment [82]. These complex effects of cannabis on WM coherence could feasibly have impacted the results in the present investigation; though we were not equipped to investigate these effects, future studies should aim to explore this further.

## Conclusions

This study has shown that acute cannabis administration reduces connectivity within resting-state networks and between brain regions associated with cognition and emotional processing. Contrary to some previous work, CBD does not appear to have any attenuating effects when combined with THC, and in some networks, the addition of CBD further reduced network connectivity. This may be due to the metabolic competition of CBD and THC leading to higher plasma THC when CBD is also present. There were no interaction effects between age group and drug treatments suggesting that adolescents do not show differential effects of cannabis compared to adults. These results therefore suggest that cannabis containing high levels of CBD may not necessarily be safer for users and that adolescent cannabis users appear to be at no greater risk than young adults with acute cannabis use, however further research is required to assess the long-term effects of cannabis, particularly past young adulthood.

### Supplementary information

Supplementary materials
