# Supplementary material for: Acute effects of different types of cannabis on young adult and adolescent resting-state brain networks
Source: Neuropsychopharmacology. 2024 May 28;49(10):1640–51. doi: 10.1038/s41386-024-01891-6 (PMC11319659; doi:10.1038/s41386-024-01891-6)
Supplement: Supplementary file 1 — Supplementary materials [file 41386_2024_1891_MOESM1_ESM.docx]

**Supplementary Materials:**

**Acute effects of different types of cannabis on adult and adolescent resting-state brain networks**

Natalie Ertl^1,2^, Tom P Freeman^3,4^, Claire Mokrysz^3^, Shelan Ofori^3^, Anna Borissova^3,5^, Kat Petrilli^4^, H. Valerie Curran^3^, Will Lawn^3,5^, *Matthew B Wall^1,2^

^1^Invicro London, Burlington Danes Building, Hammersmith Hospital, Du Cane Road, London, W12 0NN, UK

^2^Faculty of Medicine, Imperial College London, Hammersmith Hospital, Du Cane Road, London, W12 0NN, UK

^3^Clinical Psychopharmacology Unit, University College London, 1-19 Torrington Place, London, WC1E 7HB, UK.

^4^Addiction and Mental Health Group (AIM), Department of Psychology, University of Bath, Bath, UK

^5^National Addiction Centre, Institute of Psychiatry Psychology and Neuroscience, King’s College London, London, UK

***Corresponding Author:**

Matthew B. Wall

Burlington Danes Building

Hammersmith Hospital

Du Cane Road

London, W12 0NN, UK

Tel: 07801518566
[matthew.wall@imperial.ac.uk](mailto:matthew.wall@imperial.ac.uk)

**Methods:**

*Participants*

Participants had to be semi-regular cannabis users, with usage frequency between 0.5 and 3 days/week averaged over the past 3 months. This range was selected to increase the generalisability of our results, ensure participants were not extremely heavy users, likely to be tolerant to the effects of cannabis (and complete the MRI scans), and allowed for adequate recruitment. Use frequency was explicitly matched between the two age groups. Participants also had to self-report that they were able to consume approximately half a typical joint of cannabis by themselves within 20 minutes. This was to ensure that participants were experienced users and were therefore likely to tolerate the acute drug challenge well, and without adverse events. Adult users were excluded if they had had a period of three months (or more) in which cannabis was consumed at a frequency of once per week (or more) prior to the age of 18 to ensure that they had not used cannabis during this key developmental window. Participants were also in good general physical health and were not receiving treatment for any mental health condition or currently taking any prescribed psychoactive medication. Maternal education was recorded as a socioeconomic marker. Beck Depression Inventory (BDI) was recorded to index depression and control for potential differences across the age groups. Use of alcohol and other drugs was recorded for the same reason.

*MRI data acquisition*

Standard shimming was applied throughout, and ‘dummy’ scans acquired before T1 stabilization had been reached were discarded automatically by the scanner. T_2_* images were acquired using a multiband gradient echo Echo-Planar Imaging (EPI) sequence (TR = 1250 ms, echo time, TE = 30 ms, flip angle = 62°, multiband acceleration factor = 2, GRAPPA = 2, bandwidth = 1906Hz/pixel). This sequence was based on those previously documented and validated on both scanners by Demetriou et al. (2018). A total of 384 volumes were collected for each subject, with a field-of-view of 192 mm and a matrix size of 64 x 64 mm, yielding an in-plane resolution of 3 x 3 mm. Slice thickness was also 3 mm, resulting in isotropic voxels. Forty-four slices were collected using an interleaved acquisition. Phase encoding direction was anterior to posterior. The forebrain, midbrain, and hindbrain (including the cerebellum) were covered. T_1_-weighted structural images were acquired using a Magnetization Prepared Rapid Gradient Echo (MPRAGE) sequence (TR = 2300 ms, TE = 2.98 ms, flip angle = 9°, parallel imaging acceleration factor = 2), with a spatial resolution of 1 mm isotropic.

*Pre-processing*

FSL (FMRIB Software Library v6.0, Analysis Group, FMRIB, Oxford, UK), with the fMRI Expert Analysis Tool (FEAT; (Smith et al. 2004; Woolrich et al. 2001) was used to carry out all pre-processing and analyses of the fMRI data. Structural high resolution (anatomical) images were pre-processed using the fsl_anat function, which implements brain extraction, bias field correction, normalisation, and tissue segmentation. Pre-processing of the functional data consisted of head motion correction (with MCFLIRT), brain extraction (with BET) temporal filtering (100s), and spatial smoothing (6mm FWHM Gaussian kernel). The functional images were then normalised to MNI-152 (Montreal Neurological Institute) space with FNIRT (FMRIB’s non-linear registration tool), using a 10 mm warp resolution and 12 degrees of freedom. The following outcomes were inspected for each subject, to ensure quality of data: Movement estimates, including maximum estimated translation and rotation in all directions, and mean displacement. Participants were excluded if they exceed >3mm movement in any direction and >1mm mean displacement. Mean movement was then compared across age and drug groups to check for any significant differences which may bias the data.

*Head-motion*

Method: Framewise displacement was calculated by finding the difference between each timepoint, and then taking the mean for each participant. Total displacement was calculated by finding the maximum displacement and subtracting the minimum displacement. Outliers were defined as framewise displacements greater than 0.2 and 0.5, the number of these outliers was counted and compared across treatment conditions and groups.

Results:

No significant effect of drug or age were found on any of the head-motion measures. A small interaction effect was identified in the total displacement.

| **Head-motion measure** | **Drug effect** | **Age effect** | **Interaction** |
| --- | --- | --- | --- |
| Framewise displacement | F(2, 88) = 2.07, P=0.13 | F(1,44)=0.11,  P=0.74 | F(2,88)=0.009,  P=0.99 |
| Total displacement | F(2, 88) = 0.41, P=0.66 | F(1,44)=0.035,  P=0.85 | F(2,88)=3.187,  P=0.046 |
| Outliers >0.2 | F(2, 88) = 1.11, P=0.33 | F(1,44)=0.329,  P=0.56 | F(2,88)=1.957,  P=0.147 |
| Outliers >0.5 | F(2, 88) = 0.098, P=0.90 | F(1,44)=0.471,  P=0.49 | F(2,88)=1.971,  P=0.145 |

**Supplementary Table 1.** ANOVA results for head-motion parameters.

**Supplementary Figure 1**. Head-motion measures of framewise displacement (FD), total displacement (max – min), number of outliers with framewise displacement >0.2mm and >0.5mm.

*Seed Regions*

| **Seed Region** | **X** | **Y** | **Z** |
| --- | --- | --- | --- |
| Anterior Insula | (R) 38.90  (L) -36.24 | (R) 22.33  (L) 20.14 | (R) -3.38  (L) -3.84 |
| Associative striatum | (R) 23.88  (L) -23.88 | (R) 7.93  (L) 7.93 | (R) 2.07  (L) 2.07 |
| Limbic striatum | (R) 16.07  (L) -16.62 | (R) 10.88  (L) 11.97 | (R) -8.39  (L) -8.39 |
| Sensorimotor striatum | (R) -29.15  (L) -29.15 | (R) -7.64  (L) -7.64 | (R) 2.98  (L) 2.98 |
| Dorsolateral prefrontal cortex | (R) 47.54  (L) -46.63 | (R) 8.84  (L) 13.84 | (R) 28.91  (L) 29.37 |
| Hippocampus | (R) 28.06  (L) -25.34 | (R) -18.54  (L) -17.45 | (R) -16.12  (L) -17.03 |
| Posterior Cingulate Cortex | -0.22 | -51.67 | 27.09 |

**Supplementary Table 2:** Approximate centre of gravity coordinates in MNI152 standard space of the seven seed regions.


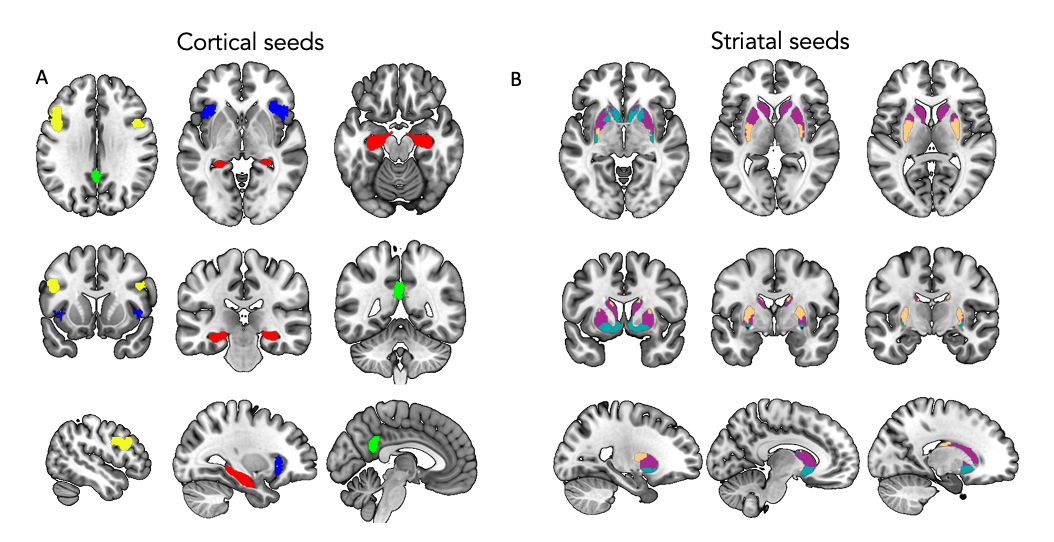


**Supplementary Figure 2:** Regions of interest used in first level analysis to define the cortical (A) and striatal (B) networks. Posterior cingulate cortex (green A) was used to define the default mode network, dorsolateral pre-frontal cortex (yellow A), was used to define the executive control network, Anterior insula (blue A) was used to define the salience network and the hippocampus (red A) was used to define the hippocampal network. The striatal networks were defined using the associative (purple B), limbic (turquoise B) and sensorimotor striatum (coral B).

*Generation of regressors*

Mean white matter (WM) and cerebro-spinal fluid (CSF) masks were also produced as part of the anatomical image processing, by FSL’s FAST algorithm. These masks were also co-registered to each subject’s functional space and thresholded at 0.5. Mean signals from these masks were extracted and included in each model as regressors of no interest to reduce the effect of noise, along with an extended set of motion parameters including temporal derivatives and quadratic versions of the six (three translations, and three rotations) basic motion parameters. The inclusion of WM and CSF regressors is similar to the CompCor approach (Behzadi et al. 2007), is a principled, robust, and effective method of reducing the influence of a range of noise sources (e.g. physiological, motion, thermal), and is useful for both resting-state (Demetriou et al. 2018; Comninos et al. 2018; Wall, Lam, et al. 2022) and task fMRI data (Thurston et al. 2022; Mills et al. 2023). Functional connectivity with each seed region was assessed using a positive contrast for the regressor of interest (the time series from each seed-region) in each model.

**Results:**


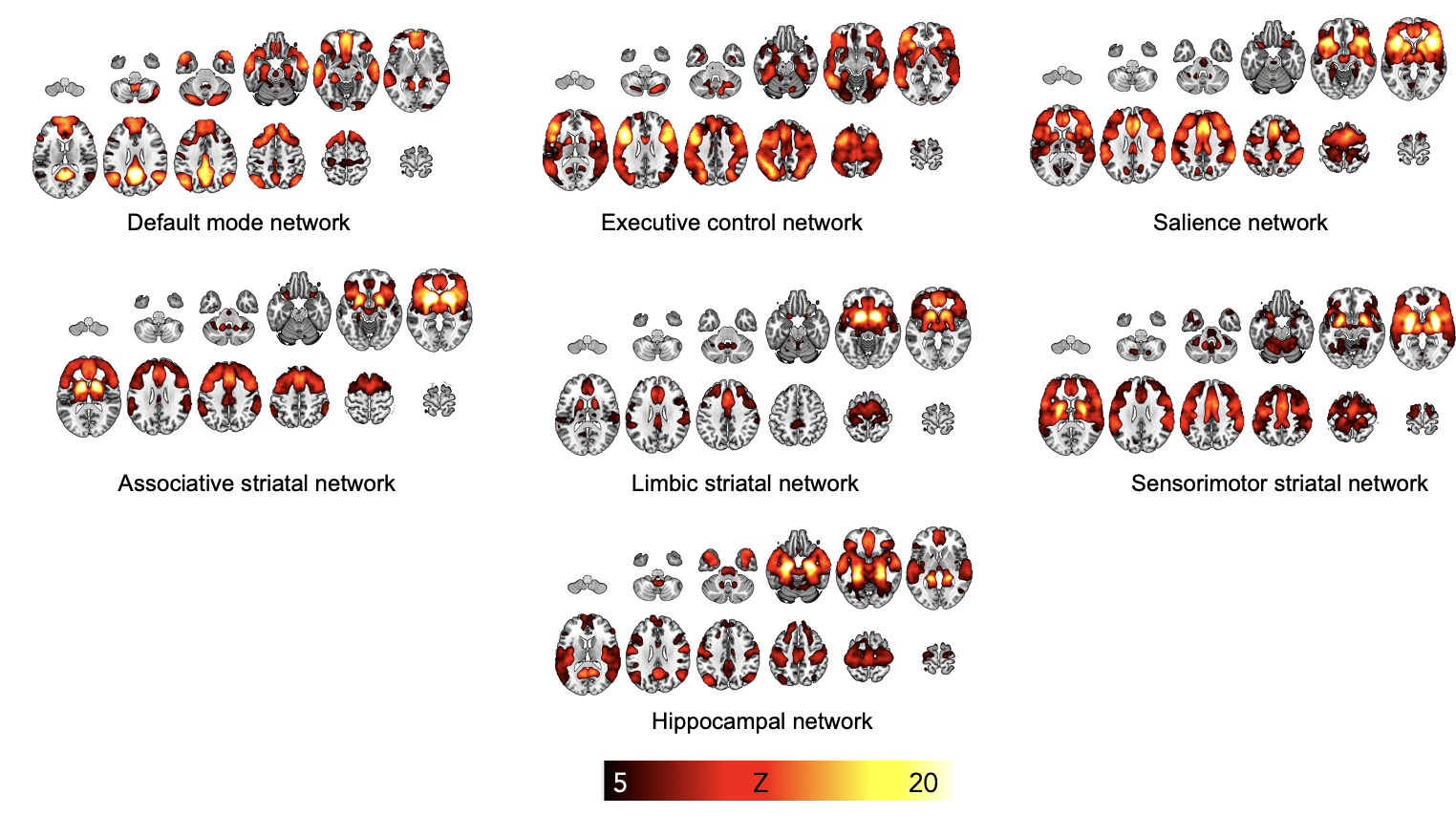


**Supplementary Figure 3.** Entire sample mean results showing networks derived from the seed regions. DMN was derived from a PCC seed, ECN was derived from a DLPFC seed, salience network was derived from an anterior insula seed. Results are cluster corrected and thresholded Z>2.3, P<0.05, N=46 (24 adults) A higher threshold of Z=5 is used here for clearer visualisation purposes.

| **Network** | **Min cluster size (voxels)** | **Z threshold (80% of Max)** |
| --- | --- | --- |
| DMN | 505 | 11.35 |
| ECN | 495 | 10.35 |
| Salience network | 451 | 11.26 |
| Associative striatum | 430 | 10.14 |
| Limbic striatum | 409 | 7.69 |
| Sensorimotor striatum | 430 | 9.42 |
| Hippocampal network | 442 | 9.03 |

**Supplementary Table 3.** Minimum cluster size required to pass cluster thresholding and 80% of the maximum Z threshold, used to threshold network maps to produce network ROIs (shown in supplementary figure 3).


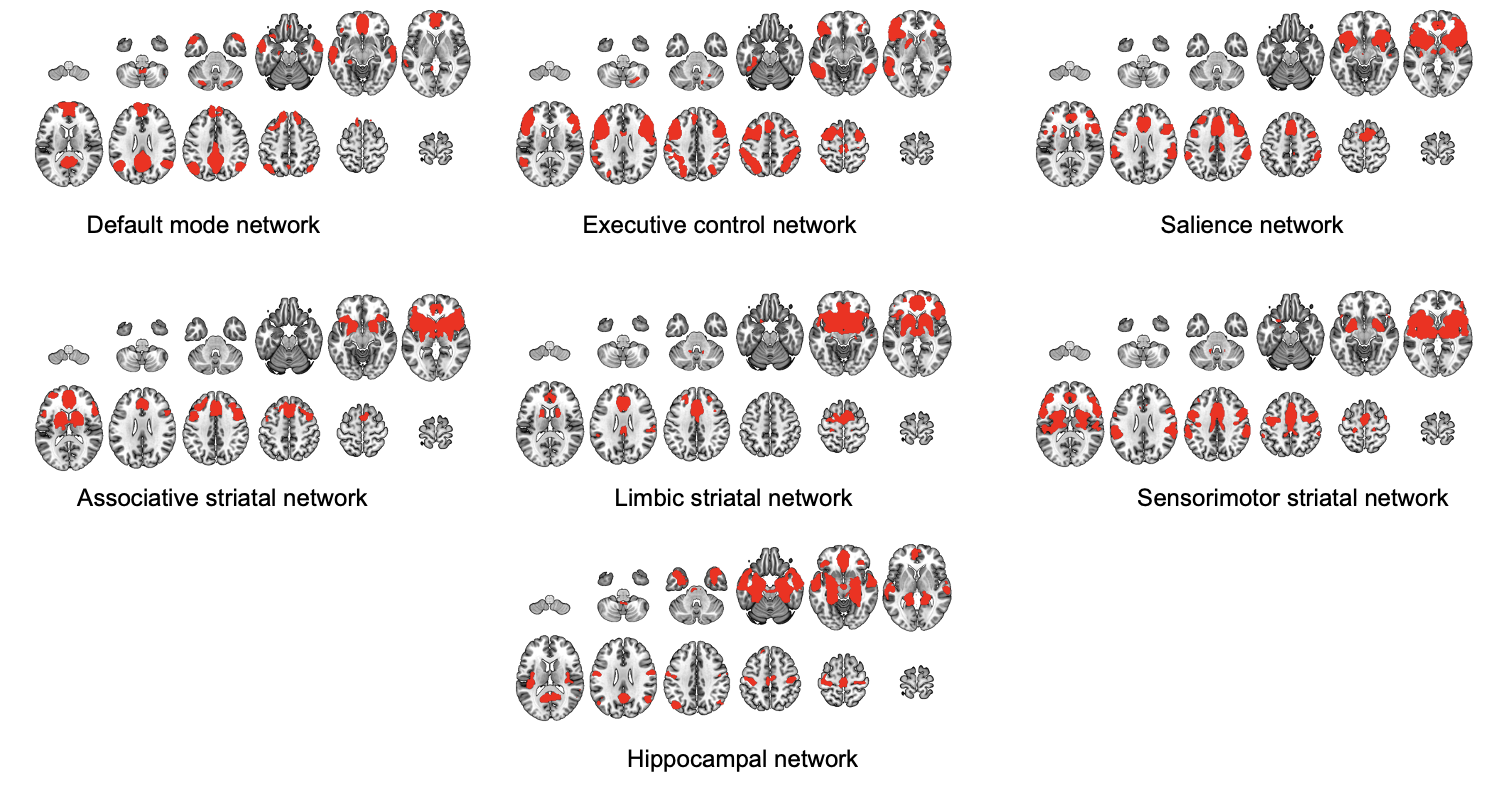


**Supplementary Figure 4.** Network masks produced from entire sample mean networks (shown in Supplementary Figure 2) thresholded at 80% of the Z max and binarised.

| Network | Drug | Effect size η² | Age | Effect size η² | Interaction |
| --- | --- | --- | --- | --- | --- |
| 1. DMN | **F[2,88]=3.97, P=0.022 *** | 0.018 | **F[1,44]=5.43, P=0.024 *** | 0.002 | F[2,88]=0.397, P=0.673 |
| 1. ECN | **F[2,88]=18.62, P<0.001 ***** | 0.123 | F[1,44]=1.33, P=0.254 |  | F[2,88]=1.33, P=0.254 |
| 1. Salience network | **F[2,88]=12.12, P<0.001 ***** | 0.076 | F[1,44]=0.253, P=0.617 |  | F[2,88]=1.51, P=0.226 |
| 1. Associative striatum | F[2,88]=2.07, P=0.133 | 0.019 | F[1,44]=3.18, P=0.081 |  | F[2,88]=0.266, P=0.767 |
| 1. Limbic striatum | **F[2,88]=16.19, P<0.001 ***** | 0.102 | F[1,44]=1.84, P=0.182 |  | F[2,88]=0.362, P=0.723 |
| 1. Sensorimotor striatum | F[2,88]=1.68, P=0.193 | 0.014 | F[1,44]=0.356, P=0.554 |  | F[2,88]=1.10, P=0.336 |
| 1. Hippocampal network | **F[2,88]=14.65, P<0.001 ***** | 0.087 | F[1,44]=0.906, P=0.347 |  | F[2,88]=0.716, P=0.491 |

**Supplementary Table 4.** Summary statistics of network ANOVA effects. Significant effects highlighted in bold, N=46 (24 adults), **P<0.05, ** P<0.01, ***P<0.001*

| **Network** | **Contrast** | **Sig** | **Main effect of drug** | |
| --- | --- | --- | --- | --- |
|  |  |  | t [df] | p |
| DMN | Placebo > THC | * | 2.56 [44] | 0.036 |
|  | Placebo > THC+CBD | * | 2.59 [44] | 0.034 |
|  | THC > THC+CBD |  | 0.002 [44] | 1 |
| ECN | Placebo > THC | ** | 3.06 [44] | 0.010 |
|  | Placebo > THC+CBD | *** | 5.50 [44] | <0.001 |
|  | THC > THC+CBD | ** | 3.45 [44] | 0.003 |
| Salience network | Placebo > THC |  | 2.20 [44] | 0.082 |
|  | Placebo > THC+CBD | *** | 4.11 [44] | <0.001 |
|  | THC > THC+CBD | ** | 3.27 [44] | 0.006 |
| Associative striatum |  |  |  |  |
|  |  |  |  |  |
|  |  |  |  |  |
| Limbic striatum | Placebo > THC | *** | 4.30 [44] | <0.001 |
|  | Placebo > THC+CBD | *** | 4.86 [44] | <0.001 |
|  | THC > THC+CBD |  | 1.49 [44] | 0.306 |
| Sensorimotor striatum |  |  |  |  |
|  |  |  |  |  |
|  |  |  |  |  |
| Hippocampal network | Placebo > THC | ** | 3.39 [44] | 0.004 |
|  | Placebo > THC+CBD | *** | 5.29 [44] | <0.001 |
|  | THC > THC+CBD |  | 1.83 [44] | 0.173 |

**Supplementary Table 5.** *Post hoc* effects of within-network connectivity with acute cannabis administration, N=46 (24 adults), **P<0.05, ** P<0.01, ***P<0.001*


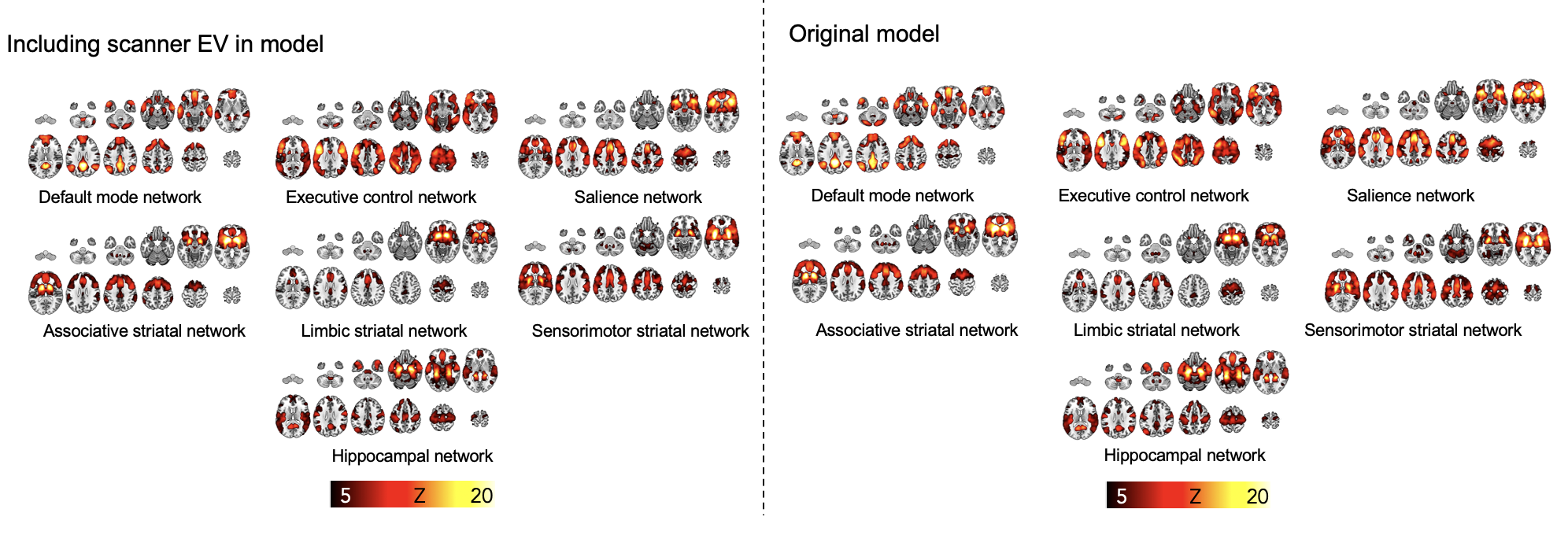


**Supplementary Figure 5**. Including the different scanners used in the study as an explanatory variable does not affect the within-network connectivity results found in the study


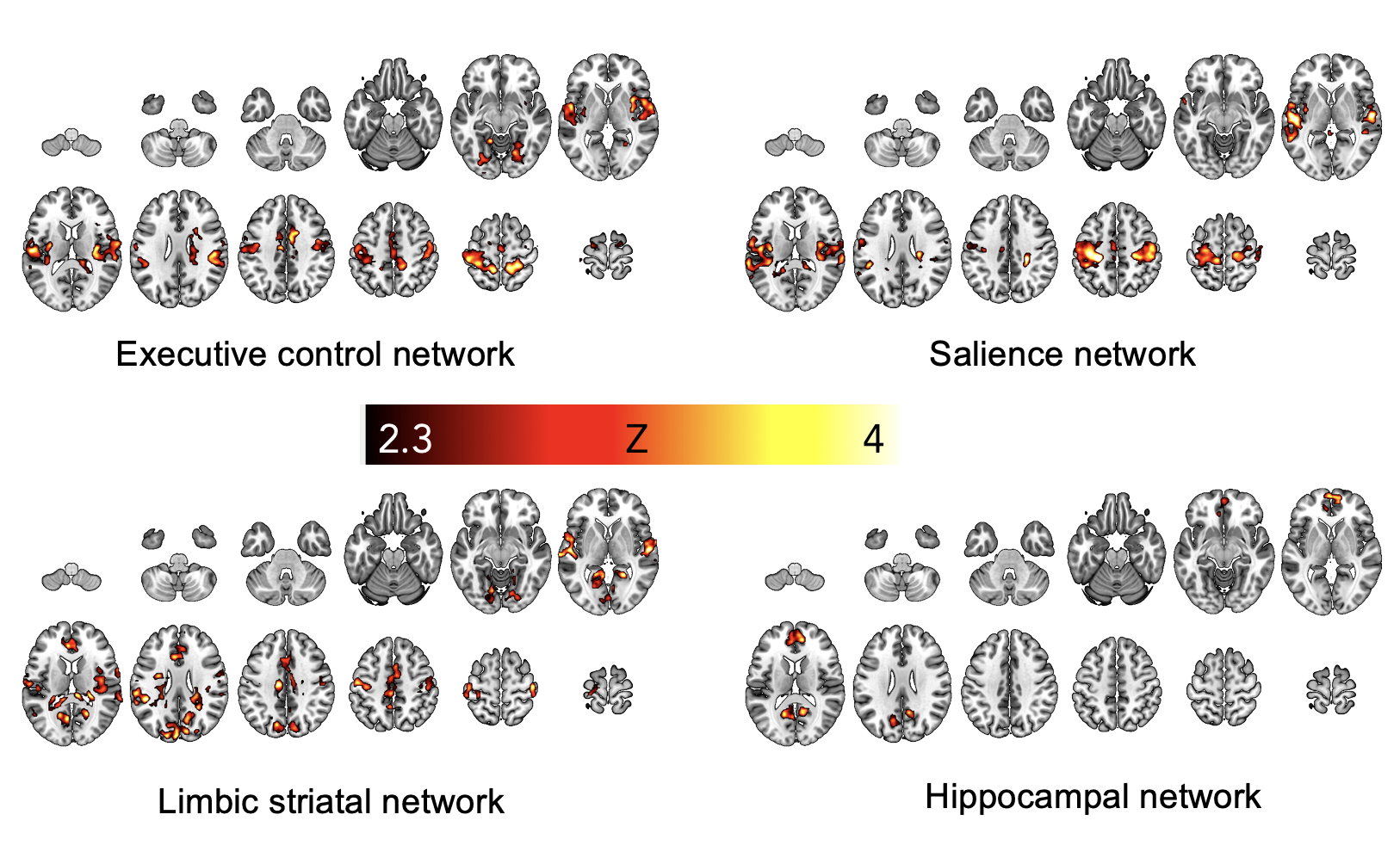


**Supplementary Figure 6.** F statistic results showing areas in which connectivity with the network is significantly altered as a result of drug treatment condition. Results are cluster corrected, *Z*>2.3, *P*<0.05, N=46 (24 adults).

| **Network** | **Min cluster size (voxels)** |
| --- | --- |
| DMN | 501 |
| ECN | 489 |
| Salience network | 445 |
| Associative striatum | 427 |
| Limbic striatum | 404 |
| Sensorimotor striatum | 482 |
| Hippocampus | 439 |

**Supplementary Table 6.**  Minimum cluster size required to pass cluster thresholding of seed-voxel whole brain analysis.

**
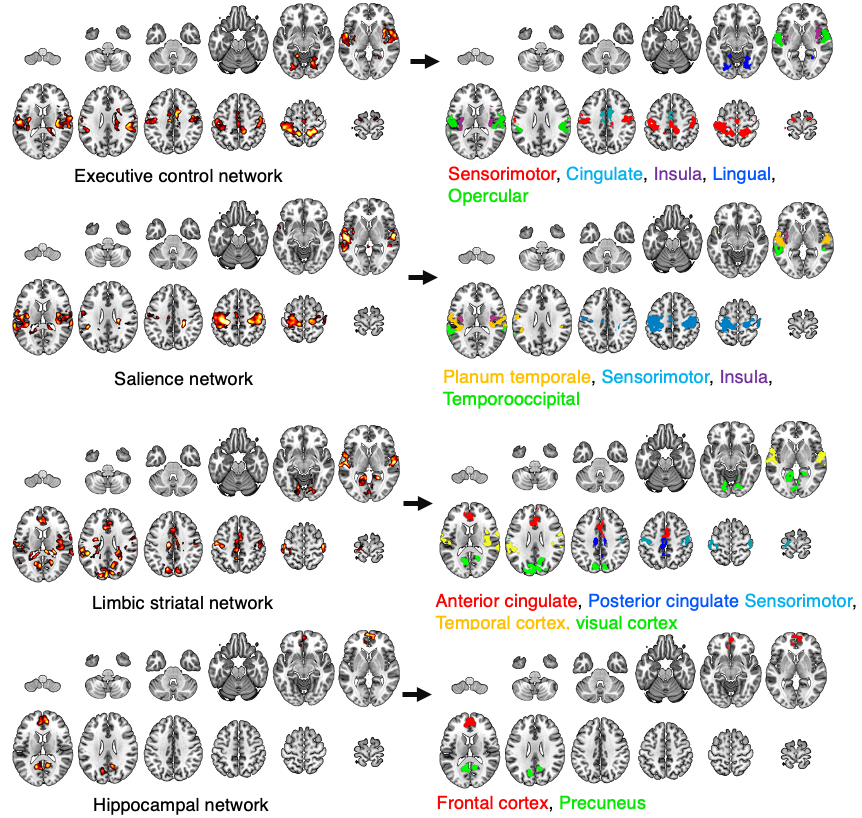
**

**Supplementary Figure 7.** Subdivision of main effect of cannabis into ROIs

**
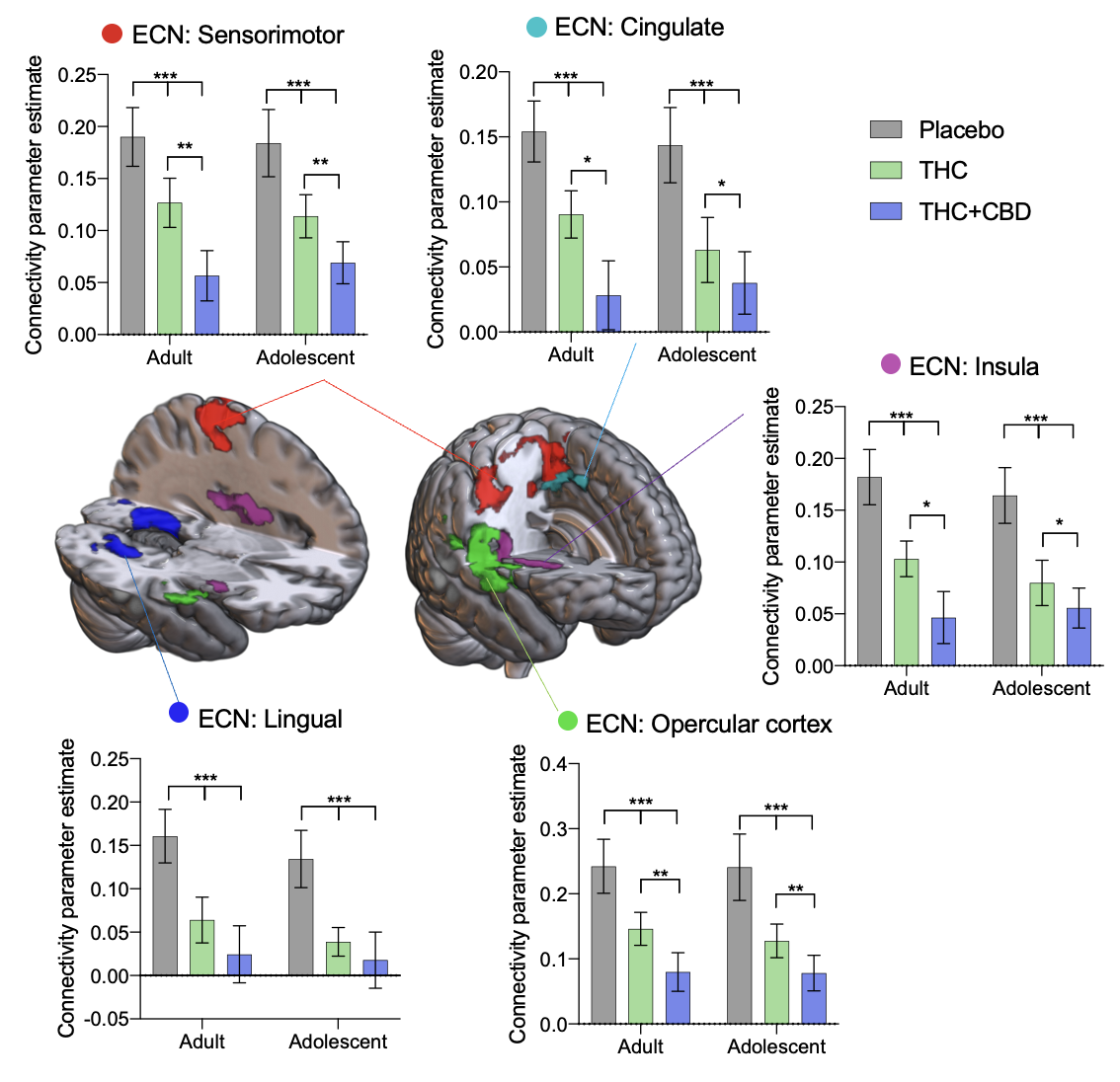
**

**Supplementary Figure 8.** Connectivity between the ECN and the sensorimotor cortex (red), midcingulate (light blue), insula (purple), opercular cortex (green), and lingual gyrus (dark blue) are significantly reduced with acute cannabis administration. Error bars show SEM, N=46 (24 adults), *P<0.001 ***, P<0.01**, P<0.05 *.*

| **ROI connectivity with the ECN** | **ANOVA** | **Effect size η²** | **‘THC’ - Placebo** | **‘THC + CBD’ -Placebo** | **‘THC+CBD’ - THC** |
| --- | --- | --- | --- | --- | --- |
| Sensorimotor cortex | F[2,88]=19.57, P<0.001 | 0.155 | t[44]=3.33, P=0.005 ** | t[44]=5.24, P<0.001 *** | t[44]=3.87, P=0.001 *** |
| Midcingulate | F[2,88]=20.84, P<0.001 | 0.147 | t[44]=4.05, P<0.001 *** | t[44]=5.71, P<0.001 *** | t[44]=2.73, P=0.024 * |
| Insula | F[2,88]=27.29, P<0.001 | 0.178 | t[44]=4.75, P<0.001 *** | t[44]=6.48, P<0.001 *** | t[44]=2.86, P=0.017  * |
| Opercular cortex | F[2,88]=18.20, P<0.001 | 0.146 | t[44]=3.58, P=0.002 ** | t[44]=5.04, P<0.001 *** | t[46]=3.12, P=0.009  ** |
| Lingual Gyrus | F[2,88]=12.11, P<0.001 | 0.132 | t[44]=3.83, P=0.001 *** | t[44]=4.19, P<0.001 *** | t[44]=1.22, P=0.447 |

**Supplementary Table 7.** ANOVA and post hoc t test results for ECN connectivity


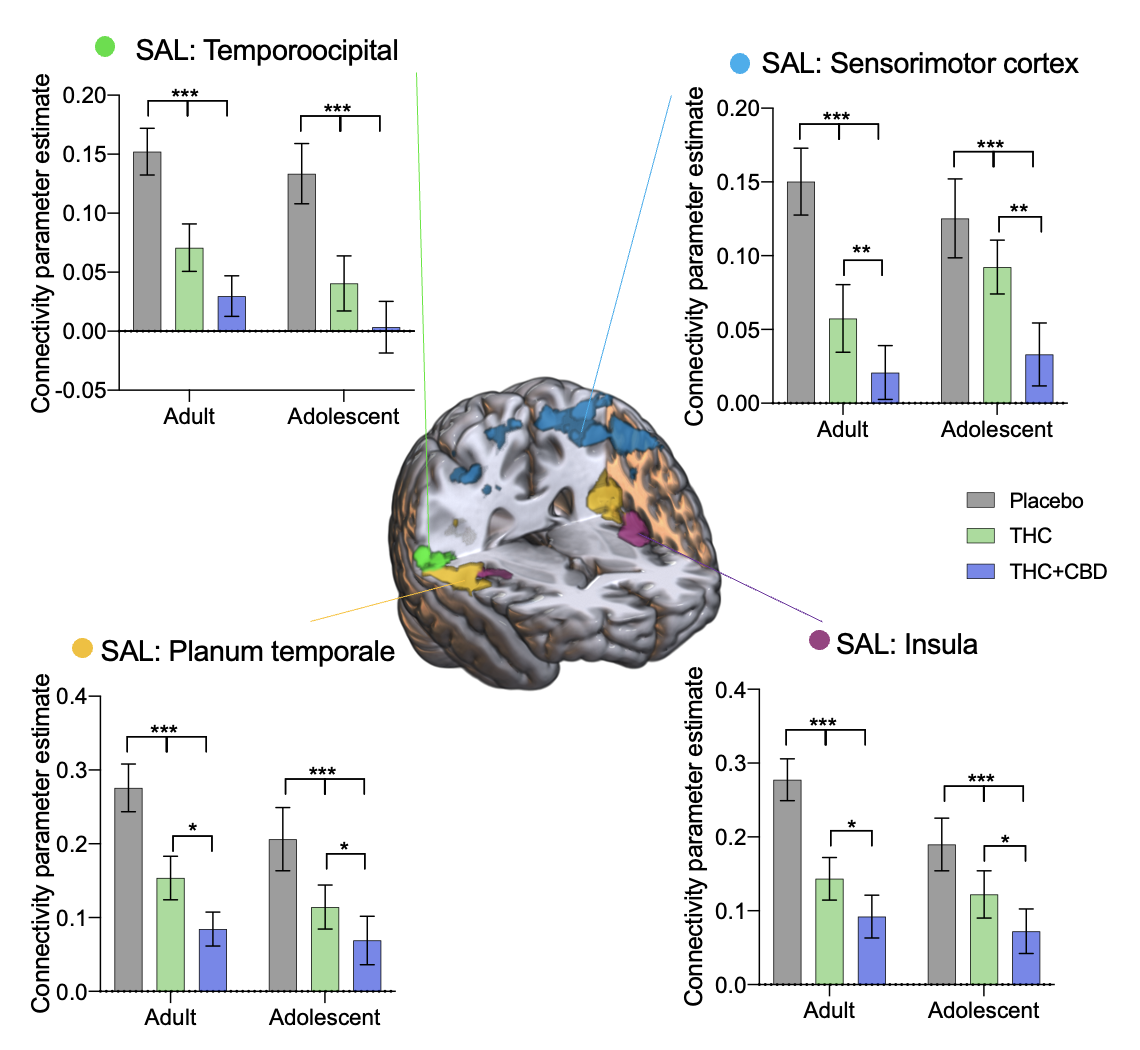


**Supplementary Figure 9.** Connectivity between the salience network and the temporo-occipital cortex (green), sensorimotor cortex (blue), planum temporale (yellow), and insula (purple) are significantly reduced with acute cannabis administration.. Error bars show SEM, N=46 (24 adults), *P<0.001 ***, P<0.01**, P<0.05 *.*

| **ROI connectivity with the salience network** | **ANOVA** | **Effect size η²** | **‘THC’ - Placebo** | **‘THC + CBD’ -Placebo** | **‘THC+CBD’ - THC** |
| --- | --- | --- | --- | --- | --- |
| Temporooccipital cortex | F[2,88]=27.05, P<0.001 | 0.215 | t[44]=4.92, P<0.001 *** | t[44]=7.14, P<0.001 *** | t[44]=4.92, P=0.073 |
| Sensorimotor cortex | F[2,88]=20.12, P<0.001 | 0.161 | t[44]=3.73, P=0.002 | [44]=3.06, P<0.001 *** | t[44]=5.61, P=0.010 ** |
| Insula | F[2,88]=20.60, P<0.001 | 0.156 | t[44]=4.06, P<0.001 | t[44]=5.57, P<0.001 *** | t[44]=2.61, P=0.032 * |
| Planum temporale cortex | F[2,88]=20.87, P<0.001 | 0.167 | t[44]=4.02, P<0.001 | t[44]=5.07, P<0.001 *** | t[44]=2.67, P=0.028 * |

**Supplementary Table 8.** ANOVA and post hoc t test results for salience network connectivity


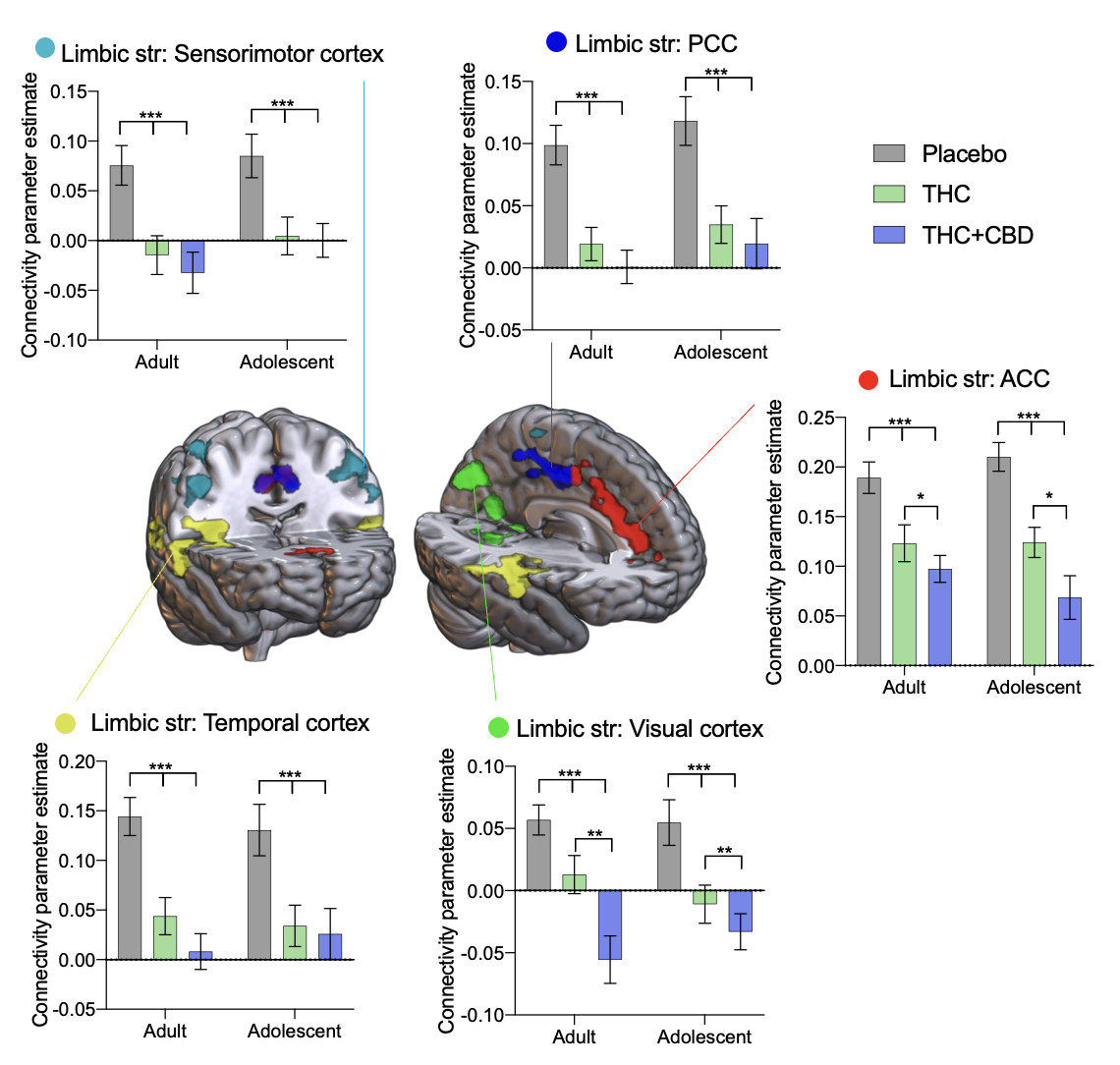


**Supplementary Figure 10.** Connectivity between the limbic striatal network and the sensorimotor cortex (light blue), posterior (dark blue) and anterior (red) cingulate, visual cortex (green), and temporal cortex (yellow) are significantly reduced with acute cannabis administration. Results are presented collapsed across age groups. Error bars show SEM, N=46 (24 adults), *P<0.001 ***, P<0.01**, P<0.05 *.*

| **ROI connectivity with the limbic striatum** | **ANOVA** | **Effect size η²** | **‘THC’ - Placebo** | **‘THC + CBD’ -Placebo** | **‘THC+CBD’ - THC** |
| --- | --- | --- | --- | --- | --- |
| Sensorimotor cortex | F[2,88]=23.96, P<0.001 | 0.175 | t[44]=5.12, P<0.001 *** | t[44]=5.66, P<0.001 *** | t[44]=0.94, P=0.614 |
| Posterior Cingulate | F[2,88]=35.84, P<0.001 | 0.237 | t[44]=5.71, P<0.001 *** | t[44]=8.04, P<0.001 *** | t[44]=1.59, P=0.259 |
| Anterior Cingulate | F[2,88]=35.84, P<0.001 | 0.270 | t[44]=5.71, P<0.001 *** | t[44]=8.04, P<0.001 *** | t[44]=2.88, P=0.017 * |
| Visual Cortex | F[2,88]=20.94, P<0.001 | 0.227 | t[44]=3.58, P=0.002 ** | t[44]=6.31, P<0.001 *** | t[44]=2.96, P=0.013 * |
| Temporal cortex | F[2,88]=26.47, P<0.001 | 0.212 | t[44]=5.57, P<0.001 *** | t[44]=5.71, P<0.001 *** | t[44]=1.66, P=0.231 |

**Supplementary Table 9.** ANOVA and post hoc t test results for limbic striatal network connectivity

| **ROI connectivity with the hippocampal network** | **ANOVA** | **Effect size η²** | **‘THC’ - Placebo** | **‘THC + CBD’ -Placebo** | **‘THC+CBD’ - THC** |
| --- | --- | --- | --- | --- | --- |
| Frontal cortex | F[2,88]=22.64, P<0.001 | 0.223 | t[44]=4.40, P<0.001 *** | t[44]=4.91, P<0.001 *** | t[44]=2.04, P=0.115 |
| Precuneus | F[2,88]=25.66, P<0.001 | 0.200 | t[44]=5.54, P<0.001 *** | t[44]=7.18, P<0.001 *** | t[44]=0.97, P=0.598 |

**
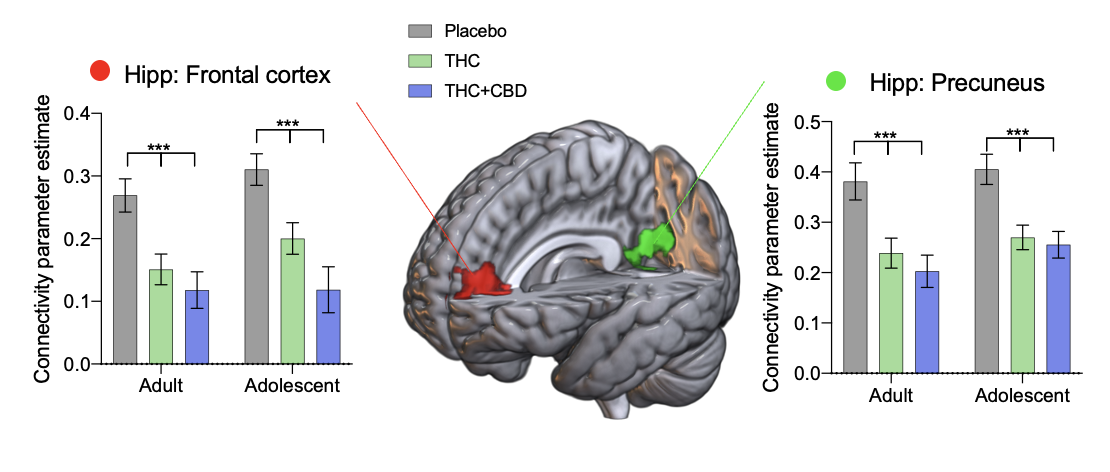
**

**Supplementary Figure 11.** Connectivity between the hippocampal network and the frontal (red) and precuneus cortex (green) is reduced with acute cannabis administration. Error bars show SEM, N=46 (24 adults), *P<0.001 ***.*

**Supplementary Table 10.** ANOVA and post hoc t test results for hippocampal network connectivity


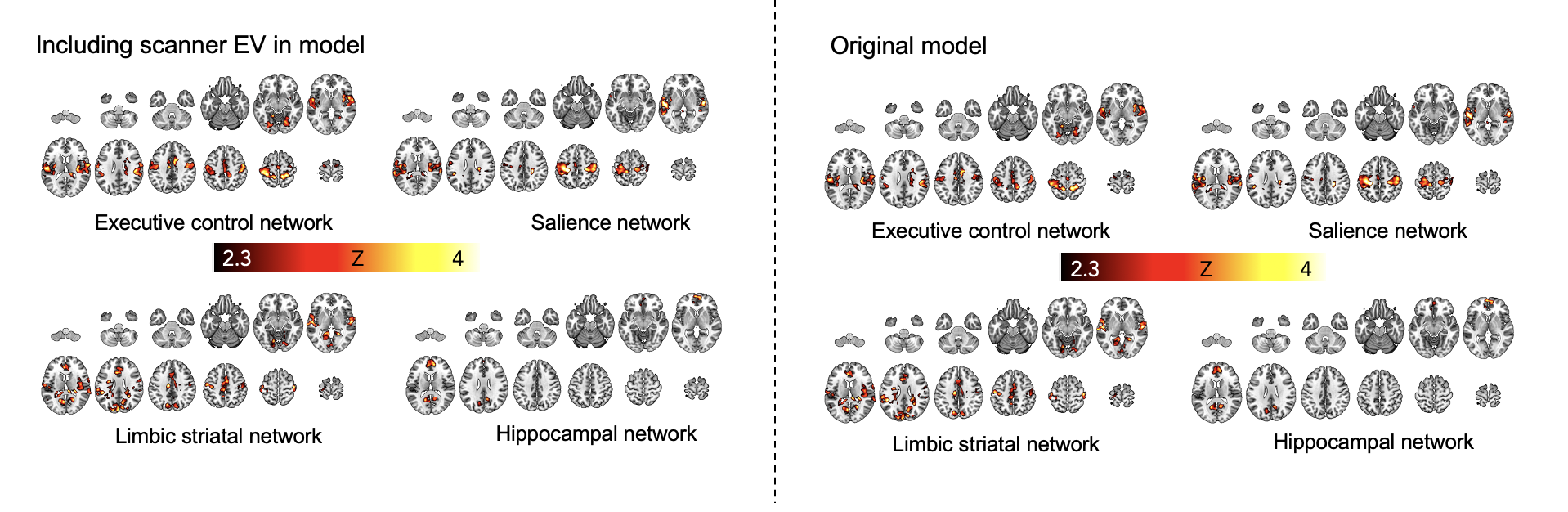


**Supplementary Figure 12.** Including the different scanners used in the study as an explanatory variable does not affect the seed-voxel connectivity results found in the study.


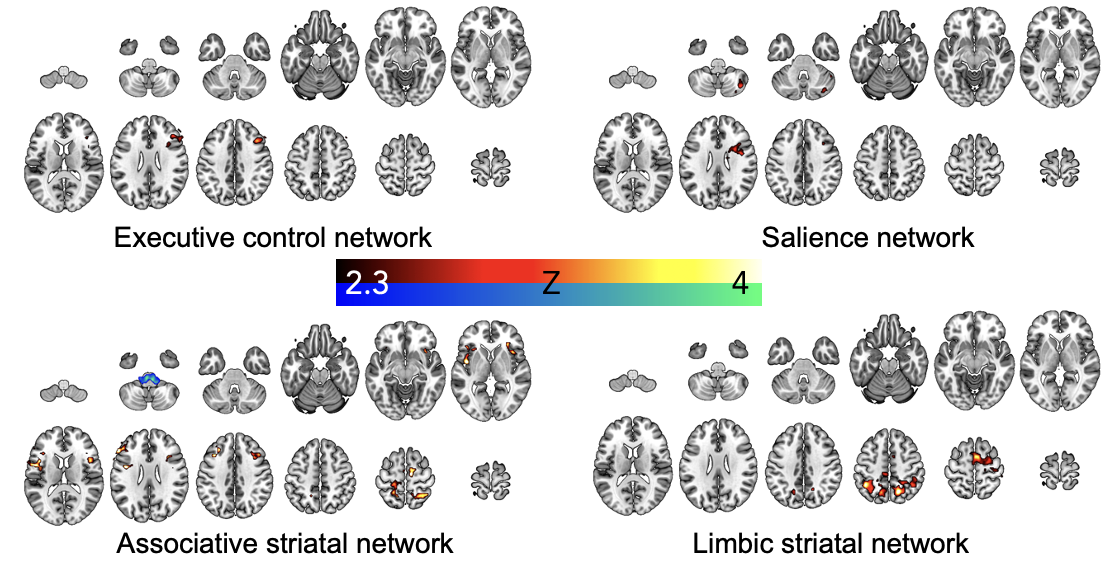


**Supplementary Figure 13.** Networks which show significant connectivity effects of age group. Areas with significantly greater connectivity in adolescent vs. adult are shown in red/yellow, areas where which have significantly greater connectivity with the network in adults v. adolescents are shown in blue/green. Results are cluster corrected, Z>2.3, P<0.05, N=46 (24 adults).
